# Artificial Intelligence in Oncology: A Comprehensive Cross-Cancer Translational Readiness Analysis Across 18 Malignancies

**DOI:** 10.3390/cancers18101543

**Published:** 2026-05-10

**Authors:** Sai Kiran Kuchana, Uday Kumar Repalle, Nikhilesh V. Alahari, Manpreet Kondamuri, Sai Kiran Manduva, Raghu Vamsi Vanguru, Sri Anjali Gorle, Suresh K. Alahari

**Affiliations:** 1 Department of Internal Medicine, Kakatiya Medical College, Warangal 506007, India; 2 Pinnamaneni Institute of Medical Sciences, Vijayawada 521286, India; 3 Department of Medicine, Tulane University, New Orleans, LA 70118, USA; 4 NRI Institute of Medical Sciences, Visakhapatnam 531162, India; 5 Kona Seema Institute of Medical Sciences, Amalapuram 533201, India; 6 Andhra Medical College, Visakhapatnam 530002, India; 7 Department of Biochemistry and Molecular Biology, LSU-LCMC Cancer Center, Louisiana State University School of Medicine, New Orleans, LA 70112, USA

**Keywords:** artificial intelligence, cancer, therapeutics, diagnosis, early detection

## Abstract

Background: Artificial intelligence (AI) is reshaping oncology at every stage of the cancer care pathway, from population-level screening through molecular diagnosis, treatment planning, and post-treatment surveillance. Despite an exponential growth in AI oncology publications exceeding 5000 peer-reviewed studies annually, a critical and persistent gap separates demonstrated algorithmic performance from genuine patient benefit. Most published evidence derives from retrospective, single-institution studies conducted in curated dataset environments that systematically differ from real-world clinical deployment conditions. This comprehensive review examines the translational maturity of AI applications across 18 major malignancies, providing an evidence-stratified, cross-cancer assessment of where AI has fulfilled, approaches, or remains far from fulfilling its transformative potential in oncological care. Methods: A structured narrative review was conducted across PubMed/MEDLINE, Embase, IEEE Xplore, and the Cochrane Library, supplemented by regulatory grey literature including FDA 510(k) decision summaries, CE Technical Files, and ClinicalTrials.gov. Search terms combined cancer site-specific terminology with AI methodology terms and translational outcome descriptors. Studies were only included if they applied an AI or machine learning methodology to a defined clinical oncological task, reported a clearly specified performance evaluation, and involved human subjects or human-derived clinical data. Evidence quality was assessed using QUADAS-2, PROBAST, and Cochrane RoB 2. A five-tier translational readiness framework, grounded in the NIH T0–T4 translational spectrum and CONSORT-AI/SPIRIT-AI guidelines, was applied a priori to enable cross-cancer comparison. A rigorous distinction was maintained between diagnostic accuracy and clinical utility, defined as demonstrated impact on clinical decision-making or patient-centered outcomes. Results: Across all 18 malignancies, AI development varied profoundly by cancer type. Breast cancer and prostate cancer (Tier 1) represent the most mature AI ecosystems, with multiple FDA-cleared tools for mammographic screening and digital pathology achieving prospective multi-institutional validation; however, randomized evidence demonstrating reduced cancer-specific mortality remains absent. Lung, hepatocellular, and melanoma AI (Tier 2) have achieved regulatory milestones but face documented performance disparities across demographic subgroups, including DermaSensor’s 20.7% specificity in primary care settings and HCC model failures in non-viral disease etiologies. Colorectal, glioma, pancreatic, and ovarian cancers (Tier 3) exhibit technical maturity without clinical clarity: colorectal CADe systems increase adenoma detection but meta-analyses of 18,232 patients across 21 RCTs fail to demonstrate improvement in advanced neoplasia detection or cancer incidence reduction. A full study-level presentation of pooled estimates, confidence intervals, and heterogeneity statistics for each cited randomized evidence base across all cancer types would extend beyond the intended scope and format of this cross-cancer narrative review. Gastric, esophageal, cervical, bladder, head and neck, and endometrial cancers (Tier 4) demonstrate promising single-institutional or geographically restricted results without multi-institutional external validation, particularly notable for cervical cancer AI’s transformative potential in low- and middle-income countries constrained by absent regulatory frameworks. Hematologic malignancies, sarcoma, and pediatric solid tumors (Tier 5) face structural barriers, workflow incompatibility in hematopathology, extreme rarity in sarcoma (>70 subtypes, <15,000 US cases annually), and irreducible ethical constraints in pediatric data governance, that cannot be resolved through algorithmic refinement alone. Conclusions: Oncological AI has not yet fulfilled its clinical promise. Across all five translational tiers, a single finding is consistent: diagnostic accuracy is not a surrogate for patient benefit. AI tools with high sensitivity and specificity have repeatedly failed to demonstrate equivalent reductions in cancer-specific mortality, overdiagnosis, or procedural harm under real-world outcome scrutiny. Simultaneously, documented performance disparities across races, ethnicity, disease etiology, and geographic setting reveal that current AI systems risk amplifying the very health inequities they are positioned to resolve. Bridging this translational gap requires three coordinated systemic shifts: regulatory frameworks mandating post-market outcome surveillance as a condition of clinical clearance; prospective trial designs measuring patient-centered endpoints rather than diagnostic concordance alone; and sustained infrastructure investment in federated data governance, demographically inclusive training datasets, and LMIC-accessible regulatory pathways. AI holds genuine potential to reduce cancer mortality on a global scale—but only if held to the evidentiary and equity standards that the stakes of oncological care demand.

## Simple Summary

Artificial intelligence (AI) is increasingly applied across the cancer care pathways, including screening, diagnosis, pathology, treatment planning, and surveillance. However, strong algorithmic performance does not necessarily translate into meaningful patient benefit in real-world clinical practice. In this review, we examine AI applications across 18 major malignancies and assess their translational readiness using a five-tier framework. We show that breast and prostate cancers have the most mature AI ecosystems, while many other cancer types remain limited by inadequate external validation, uncertain clinical utility, poor generalizability, and insufficient prospective outcome data. Across all tiers, a consistent finding emerges—diagnostic accuracy alone is not a surrogate for improved patient-centered outcomes. This review highlights the need for prospective validation, equitable dataset development, and stronger evidence linking AI tools to real-world clinical benefit.

## 1. Introduction

### 1.1. The Global Cancer Burden and the Imperative for Technological Innovation

Cancer remains one of the foremost public health challenges of the twenty-first century. According to the Global Cancer Observatory, approximately 19.3 million new cancer cases were diagnosed worldwide in 2022, with an estimated 9.7 million deaths attributable to malignant disease in the same year [1]. Projections suggest that annual incidence will surpass 28 million cases by 2040, driven by an aging global population, rising prevalence of modifiable risk factors including obesity and tobacco use, and improved diagnostic ascertainment in previously under-surveilled regions [2]. Against this epidemiological backdrop, the emergence of artificial intelligence (AI) as a transformative computational paradigm represents one of the most consequential developments in contemporary oncology. As observed in a review, AI is no longer science fiction, it is a reality with which physicians and healthcare providers must make friends and learn how to co-exist [3]. In oncology specifically, this co-existence has accelerated from a theoretical aspiration to a clinical imperative, reshaping how malignancies are detected, classified, staged, and treated [4].

Artificial intelligence is broadly defined as a computer system capable of performing tasks that normally require human intelligence, including visual perception, pattern recognition, clinical decision-making, and language interpretation [5]. Unlike conventional computer programs, which operate through fixed, programmer-defined coding sequences, AI systems work through algorithms: adaptive rule sets that allow machines not merely to execute predetermined instructions but to learn, generalize, and improve performance with exposure to new data [3]. This fundamental distinction, between static coding and adaptive algorithmic intelligence, explains why AI can perform tasks in oncology that exceed the practical capacity of even highly trained clinicians, including the simultaneous integration of imaging, genomic, pathological, and clinical data streams at a scale and speed that no human expert can match [6]. The power of AI in cancer care derives precisely from this capacity: to absorb vast quantities of heterogeneous data, detect non-obvious patterns within it, and translate those patterns into clinically actionable outputs, a capability that becomes increasingly essential as the volume and complexity of oncological data continue to grow exponentially [7].

### 1.2. Components of Artificial Intelligence: A Framework for Oncological Application

Understanding the distinct components of AI, and how each map onto specific oncological tasks, is prerequisite to evaluating the translational evidence base. Just as Kishore and Pinjala (2024) outlined the hierarchical structure of AI components from machine learning through deep learning to neural networks and generative models, the present review applies this framework explicitly to cancer medicine, tracing how each AI paradigm has been deployed, validated, and where applicable, clinically implemented across 18 malignancies [3].

AI encompasses several distinct but interrelated computational paradigms, each with specific properties and oncological applications. Machine learning (ML) constitutes the foundational layer: a self-learning system in which algorithms analyze input data, detect statistical patterns, and iteratively refine predictive models without requiring explicit re-programming by human operators [8]. Deep learning (DL) represents a specialized and substantially more powerful subset of machine learning, distinguished by its use of artificial neural networks (ANNs) with multiple processing layers, the “depth” of which enables it to extract hierarchical representations from raw, high-dimensional data including medical images, genomic sequences, and digitized histology slides [9]. Neural networks themselves are computational architectures modeled on the organizational principles of the human brain and nervous system, in which interconnected layers of artificial “neurons” process input signals in parallel and transmit weighted outputs to subsequent layers until a final prediction or classification is generated [10]. Generative AI, a more recently matured subfield, is capable of producing entirely new data content, including synthetic medical images, augmented training datasets, and clinical text, by learning the statistical distributions underlying its training corpus, a capability with significant implications for addressing data scarcity in rare oncological conditions [11]. Together, these interconnected AI paradigms constitute the computational toolkit through which the field of oncology is fundamentally reconstituted (Table 1).

#### 1.2.1. Machine Learning: The Algorithmic Foundation of Oncological AI

Machine learning, as the overarching framework within which most oncological AI applications are developed, operates through three principal learning paradigms. In supervised learning, the most widely deployed approach in clinical AI, algorithms are trained on labeled datasets in which input data (imaging features, genomic measurements, clinical variables) are paired with known outcome labels (malignant versus benign, responder versus non-responder, high-risk versus low-risk), enabling the model to learn associations that generalize to new, unlabeled cases [12]. Supervised ML algorithms applied in oncology include support vector machines (SVMs), gradient boosting classifiers such as XGBoost and LightGBM, random forest ensembles, and logistic regression models, each with distinct strengths in handling structured tabular clinical and genomic data. In unsupervised learning, algorithms identify inherent structure within unlabeled datasets, discovering patient subgroups, molecular subtypes, or imaging phenotypes without prior outcome annotation, an approach particularly valuable in exploratory cancer biology and biomarker discovery [13]. Semi-supervised and self-supervised learning approaches, which leverage large quantities of unlabeled data augmented by smaller labeled corpora, have gained substantial traction in oncology given the chronic scarcity of expert-annotated training datasets relative to the volumes of raw clinical data generated by modern healthcare systems. Across all these paradigms, the common theme is adaptation: ML models improve with exposure to data, making them fundamentally different from the static diagnostic algorithms embedded in previous generations of clinical decision support tools.

#### 1.2.2. Deep Learning and Convolutional Neural Networks: The Engine of Imaging AI

Deep learning, and in particular convolutional neural networks (CNNs), has driven the most clinically impactful advances in oncological AI to date. A CNN is a class of deep neural network architecturally optimized for processing grid-structured data, including two-dimensional medical images (mammograms, CT slices, H&E-stained pathology whole slide images) and three-dimensional volumetric imaging datasets, through the application of hierarchical convolutional filter operations that progressively extract features of increasing semantic complexity, from low-level edge and texture detectors in early layers to high-level lesion morphology representations in deeper layers [14]. The clinical significance of CNNs in oncology was first demonstrated with landmark force in dermatology and radiology: a CNN trained on 129,450 clinical images classified skin cancers with dermatologist-level accuracy, while studies applying CNNs to mammographic imaging established sensitivity and specificity metrics meeting or exceeding radiologist performance across reader studies enrolling tens of thousands of patients [15]. In the context of pathology, CNNs process digitized whole slide images (WSIs) at resolutions encompassing billions of pixels per slide, detecting malignant cells, quantifying proliferation indices, predicting molecular subtype from morphological features, and measuring spatial relationships within the tumor microenvironment, tasks that collectively represent a revolution in diagnostic pathology. The architectures most frequently applied in oncological imaging AI include ResNet (Residual Networks), DenseNet (Dense Convolutional Networks), EfficientNet, and Vision Transformers (ViT), each offering different trade-offs between computational efficiency and feature extraction depth that influence their suitability for specific cancer imaging modalities [16].

#### 1.2.3. Recurrent Neural Networks, Transformers, and Large Language Models

Beyond convolutional architectures designed for spatial image data, recurrent neural networks (RNNs) and their more advanced variants, Long Short-Term Memory networks (LSTMs) and Gated Recurrent Units (GRUs), have been applied to sequential and time-series oncological data, including longitudinal biomarker trajectories, electronic health record (EHR) temporal sequences, and serial imaging surveillance datasets [17]. RNNs process data in ordered temporal sequences, allowing them to model how clinical variables evolve over time, a property directly relevant to cancer surveillance, treatment response monitoring, and recurrence prediction [18]. More recently, transformer-based architectures, originally developed for natural language processing tasks, have been adapted for both imaging and clinical text applications in oncology [19]. Transformer models process entire input sequences simultaneously through multi-head self-attention mechanisms, capturing long-range dependencies in data that RNNs process only locally, enabling more powerful representations of both imaging content and clinical narrative text. Large language models (LLMs), which are transformer-based models trained on massive corpora of biomedical literature and clinical documentation, represent an emerging application in oncology, with demonstrated capacity for pathology report summarization, clinical trial eligibility screening, and structured data extraction from unstructured oncology notes [20]. The integration of vision transformers, language models, and multimodal architectures capable of jointly processing imaging and text data represents the frontier of AI development in oncological clinical decision support [21] (Table 2).

#### 1.2.4. Radiomics: Extracting the Molecular Phenotype from Medical Images

**Figure 1:**
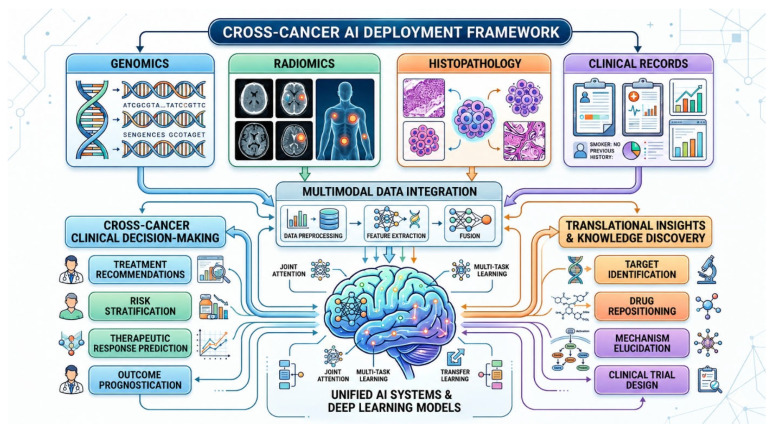
Cross-cancer AI deployment framework. A conceptual framework showing how multimodal data sources, including genomics, radiomics, histopathology, and clinical records, can be integrated into unified AI systems to support cross-cancer clinical decision-making and translational insights.

Radiomics constitutes a distinct but deeply interconnected AI methodology that extracts large numbers of quantitative features, describing shape, texture, intensity distribution, and spatial heterogeneity, from standard-of-care medical imaging modalities including CT, MRI, PET, and ultrasound, with the goal of capturing tumor biological characteristics that are imperceptible to visual radiological assessment [22,23]. A typical radiomic analysis pipeline involves image acquisition, tumor segmentation (increasingly automated through deep learning-based convolutional segmentation architectures such as U-Net), feature extraction (generating hundreds to thousands of quantitative descriptors per imaging volume), feature selection (applying dimensionality reduction and regularization methods to identify features with stable discriminative value), and predictive model construction using machine learning classifiers [24]. The clinical hypothesis underlying radiomics is that imaging phenotypes, the macroscopic spatial patterns visible on CT or MRI, encode underlying tumor genomic and microenvironmental characteristics through the physical processes of tumor growth, angiogenesis, necrosis, and invasion that determine imaging appearance [25]. This hypothesis, termed “radiogenomics” when explicitly linking imaging features to genomic alterations, has been validated across multiple cancer types: radiomic signatures derived from pretreatment CT imaging have been shown to correlate with EGFR mutation status in non-small cell lung cancer, IDH mutation in glioma, microsatellite instability in colorectal cancer, and HER2 amplification in breast cancer, among many other molecular associations [26] (Figure 1). Critically, radiomics offers the prospect of non-invasive, whole-tumor molecular phenotyping that captures spatial heterogeneity inaccessible to single-site biopsy sampling, a methodological advantage with direct clinical relevance given the documented prognostic and predictive importance of intratumoral heterogeneity across oncological contexts [27].

#### 1.2.5. Federated Learning, Generative AI, and Foundation Models

Several additional AI methodologies are reshaping the translational landscape of oncological AI in ways that extend beyond the clinical accuracy of individual predictive models [28]. Federated learning is a distributed machine learning framework in which algorithms are trained collaboratively across multiple institutions without requiring the transfer of patient-level data to a central repository, instead, only model parameter updates (gradients) are shared and aggregated centrally, with raw clinical data remaining within institutional firewalls [29]. This architecture addresses the most fundamental structural barrier to large-scale oncological AI development: the regulatory, ethical, and logistical constraints that prevent centralized pooling of sensitive patient data across hospitals, health systems, and national boundaries. In oncology, federated learning has enabled multi-center model training for breast cancer neoadjuvant response prediction, prostate cancer Gleason grading, and lung nodule risk stratification without requiring data centralization, methodological advances with direct implications for equitable AI development that does not concentrate computational power in institutions with pre-existing data infrastructure advantages [30]. Generative adversarial networks (GANs), which consist of a generator network trained to produce realistic synthetic data and a discriminator network trained to distinguish synthetic from real samples, have been applied in oncology to augment limited training datasets, generating synthetic CT images, whole slide image patches, and genomic profiles that preserve statistical properties of real data without exposing protected patient information [11]. Foundation models, large-scale AI models pre-trained on broad, multi-domain datasets and then fine-tuned for specific clinical tasks, represent the emerging frontier of generalizable oncological AI, with models such as CONCH (for pathology) and BioViL-T (for radiology) demonstrating capacity to perform multiple cancer-related tasks from a single pre-trained backbone without requiring task-specific architecture redesign [31]. The convergence of these methodological innovations, federated training, synthetic augmentation, and foundation model pre-training, is progressively dismantling the data scarcity and generalizability barriers that have historically constrained oncological AI translation.

### 1.3. AI Across the Cancer Care Pathway

**Figure 2:**
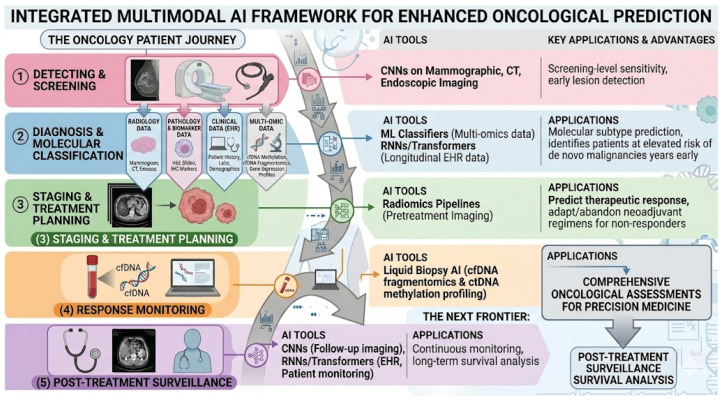
Multimodal AI integration in oncology [32,33,34,35]. Framework demonstrates how radiology, pathology, clinical data, and multi-omics information can be combined through multimodal AI models to improve diagnostic and prognostic prediction.

The operational application of these AI tools in oncology follows the clinical pathway of cancer management: detection and screening, diagnosis and molecular classification, staging and treatment planning, response monitoring, and post-treatment surveillance [6]. At each stage, different AI architectures offer distinct advantages. CNNs applied to mammographic, CT, and endoscopic imaging have demonstrated screening-level sensitivity for early lesion detection [32]. ML classifiers trained in multi-omics data enable molecular subtype prediction that guides systemic therapy selection. Radiomics pipelines applied to pretreatment imaging predict therapeutic response before treatment initiation, allowing neoadjuvant regimens to be adapted or abandoned in predicted non-responders [33]. RNNs and transformer models applied to longitudinal EHR data identify patients at elevated risk of developing de novo malignancies years before clinical presentation [34]. Liquid biopsy AI applied to cfDNA fragmentomics and ctDNA methylation profiling enables non-invasive disease monitoring between imaging assessments, potentially detecting molecular recurrence months before radiographic relapse [35]. The integration of these modality-specific AI tools into unified, multimodal clinical decision support systems, in which imaging, pathology, genomics, and clinical narrative data are jointly processed to generate comprehensive oncological assessments, represents the next developmental frontier [36] (Figure 2). However, as this review demonstrates, the path from technical feasibility to genuine patient benefit is neither linear nor uniform across cancer types.

### 1.4. The Translational Gap and the Purpose of This Review

**Figure 3:**
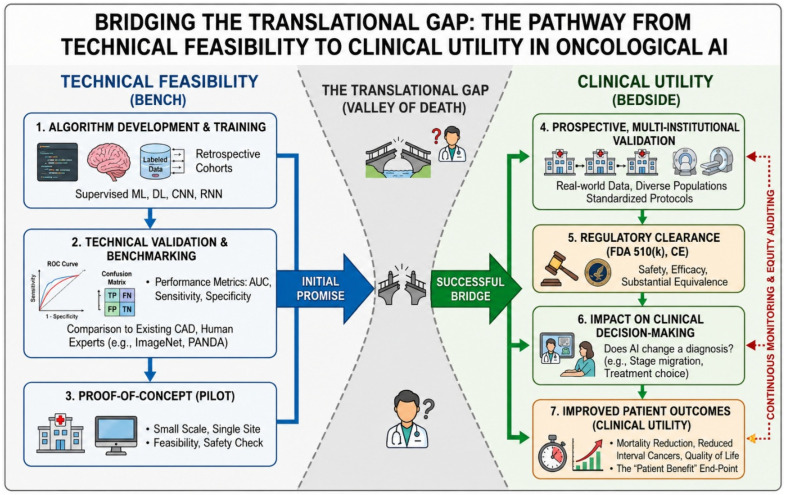
Bridging the translational gap in oncology AI. This schematic illustrates the progression from algorithm development and retrospective validation to prospective testing, clinical integration, and patient-centered outcomes, highlighting the gap between technical performance and real-world utility.

Despite the exponential growth of AI oncology publications, exceeding 5000 peer-reviewed studies annually as of 2023, a critical and persistent gap separates demonstrated algorithmic performance from evidence-based clinical impact. Retrospective technical validation studies, which constitute the overwhelming majority of published AI oncology research, evaluate AI performance in controlled, curated dataset environments that systematically differ from real-world clinical deployment conditions (Figure 3) [10]. In real-world settings, performance is degraded by imaging protocol heterogeneity, missing data, population demographic mismatch relative to training cohorts, and workflow integration challenges that affect the fidelity of AI output interpretation [37]. Regarding AI in healthcare broadly, algorithms trained on datasets that reflect existing biases will incorporate those blind spots, a concern acutely realized in oncological AI, where documented performance disparities across race, ethnicity, skin tone, and geographic population have been identified in breast cancer screening, melanoma detection, and cervical cancer AI tools deployed without adequate demographic representation in training data [3]. Most fundamentally, improved diagnostic accuracy does not automatically translate to improved patient outcomes: a lesson taught repeatedly in oncology, from PSA-based prostate cancer screening to first-generation mammographic CAD, where detection improvements failed to reduce cancer-specific mortality at the population level. The present comprehensive review is designed to characterize the translational readiness (Table 3) of AI across 18 major malignancies with explicit reference to these lessons, providing a rigorous, evidence-stratified assessment of where AI has fulfilled, where it approaches, and where it remains far from fulfilling its transformative potential in oncological care.

The application of these AI methodologies across oncological domains spans the full spectrum of the cancer care pathway: from population-level risk stratification and early detection through screening program augmentation, to histopathological diagnosis and molecular subtype classification, to treatment planning and response prediction, to post-treatment surveillance and recurrence detection. The areas of cancer medicine most substantially impacted by AI to date are those with established digital data infrastructure, radiology, digital pathology, and genomics, where large annotated datasets have been accumulated over decades, creating the training corpora necessary for supervised deep learning models to achieve clinical-grade performance [3]. However, as the current review demonstrates, the maturity of AI translation varies profoundly across cancer types, reflecting not only the availability of training data but the biological complexity of the disease, the regulatory pathway for AI device approval, the equity of representation in training populations, and most critically, whether algorithmic performance has been validated against the patient-centered outcomes that constitute genuine clinical benefit. The present comprehensive review systematically characterizes the translational readiness of AI applications across 18 major malignancies, stratified into five evidence-based tiers ranging from near-clinical integration to very limited translation, with explicit attention to the AI methodologies deployed, the clinical questions addressed, the translational barriers identified, and the development priorities that must be achieved before AI can fulfill its transformative potential in each oncological domain.

## 2. Materials and Methods

### 2.1. Study Design

This study was conducted as a comprehensive narrative review with a structured literature search, designed to examine the translational maturity of artificial intelligence across 18 major cancer types. The review was designed to assess the full translational arc of AI in oncology, from algorithmic development through regulatory approval to real-world clinical deployment with particular attention to clinical utility, equity considerations, and barriers to implementation. The review was designed to examine how artificial intelligence has advanced across different oncologic settings with respect to translational maturity, real-world clinical applicability, and implementation barriers. Accordingly, the review was intentionally designed to preserve breadth across 18 malignancies to enable cross-cancer comparisons of translational maturity, shared evidentiary limitations, and recurring barriers to clinical implementation.

### 2.2. Search Strategy

A systematic literature search was conducted across PubMed/MEDLINE, Embase, IEEE Xplore, and the Cochrane Library. Grey literature sources included FDA 510(k) and De Novo decision summaries, CE Technical Files, ClinicalTrials.gov, and the WHO International Clinical Trials Registry Platform. Search terms combined cancer-site-specific terminology with AI methodology terms (e.g., “machine learning,” “deep learning,” “neural network,” “radiomics,” “natural language processing,” “liquid biopsy”) and translational outcome terms (e.g., “clinical validation,” “regulatory approval,” “prospective study”). No date restriction was applied. Searches were last updated in 10 February 2026.

### 2.3. Inclusion Criteria

Studies were eligible for inclusion if they met all the following criteria:

- Reported the application of an AI or ML methodology to a clinical oncological task across any of the 18 cancer types under review;
- Included a clearly defined performance evaluation (e.g., diagnostic accuracy, prognostic prediction, treatment response, segmentation quality, or risk stratification);
- Provided sufficient methodological detail to assess study design, dataset characteristics, and validation approach;
- Were published in peer-reviewed journals or represented regulatory-grade evidence (FDA, CE, PMDA submissions);
- Reported studies involving human subjects or human-derived clinical data.

### 2.4. Exclusion Criteria

Studies were excluded if they met any of the following criteria:

- Preclinical or purely computational studies with no clinical data or clinical translation pathway described;
- Conference abstracts, editorials, and opinion pieces without original data;
- Studies limited to a single modality benchmark with no independent external validation and no clinical comparator;
- Duplicate publications reporting identical cohorts without additional data or analysis;
- Studies that did not report sufficient performance metrics to enable quality assessment.

### 2.5. Data Extraction

Data were extracted independently by at least two reviewers using a standardized extraction template. Extracted fields included AI methodology class, data modality, training and validation dataset characteristics (sample size, institution count, geographic and demographic composition), validation approach, performance metrics, regulatory status, equity-stratified subgroup data, and clinical utility evidence. Discrepancies were resolved through structured discussion and reference to primary sources.

### 2.6. Quality Assessment

Methodological considerations relevant to diagnostic accuracy studies, prediction model studies, and randomized trial evidence were interpreted with reference to established frameworks, including QUADAS-2, PROBAST, and Cochrane RoB 2. However, a formal study-level risk-of-bias tabulation was not performed, as the objective of this review was narrative cross-cancer translational synthesis rather than systematic evidence grading.

### 2.7. Translational Readiness Classification

**Figure 4:**
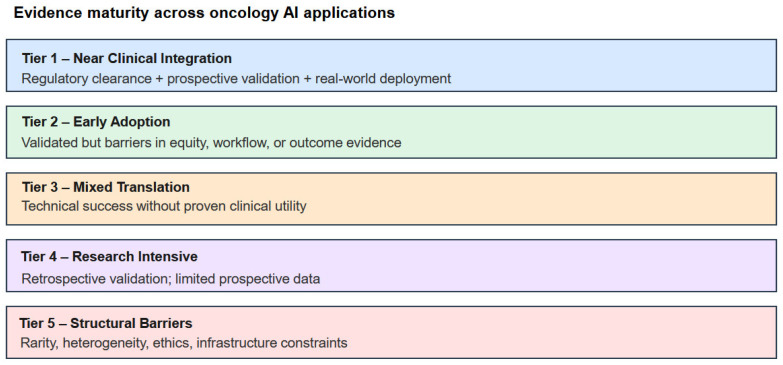
Five-tier translational readiness framework for oncology AI. Classification framework used in this review to categorize cancer types according to the maturity of AI development and clinical implementation, from near-clinical deployment to structural barriers limiting progress.

A five-tier translational readiness framework was applied a priori to enable cross-cancer comparison, grounded in the NIH T0–T4 translational spectrum and the CONSORT-AI and SPIRIT-AI reporting guidelines. Tiers ranged from Tier 1 (Near-Clinical Integration: regulatory approval achieved, prospective multi-institutional validation, documented real-world deployment) to Tier 5 (Limited Translation: predominantly single-institution feasibility evidence with fundamental structural barriers to clinical deployment). Tier assignments were made by consensus review with documented rationale for each cancer type (Figure 4 and Table 3). Future work may further operationalize and validate this framework through consensus-based or quantitative methodological approaches.

## 3. Results and Discussion

The provided graphic (Figure 4) outlines a five-tier framework for assessing evidence maturity across oncology AI applications, ranging from experimental stages to full clinical implementation. The most advanced level, Tier 1 (Near Clinical Integration), includes applications that have secured regulatory clearance, undergone prospective validation, and achieved real-world deployment. Tier 2 (Early Adoption) represents validated technologies that still face hurdles related to equity, workflow integration, or outcome evidence, while Tier 3 (Mixed Translation) describes tools with technical success but no proven clinical utility. The lower stages consist of Tier 4 (Research Intensive), characterized by retrospective validation and limited prospective data, and Tier 5 (Structural Barriers), where progress is hindered by challenges such as rarity, ethics, and infrastructure constraints.

### 3.1. Tier 1: Near-Clinical Integration: High-Data, Standardized Cancers

Breast and Prostate cancers represent the most advanced stage of AI translation in oncology, supported by high-incidence disease burden, standardized diagnostic workflows, and large, well-annotated datasets. These malignancies benefit from established screening programs, digital imaging infrastructure, and regulatory-cleared AI tools that have undergone multi-institutional validation. However, near-clinical integration does not equate to proven outcome benefit. In this tier, the central question has shifted from whether AI can perform accurately to whether it meaningfully improves patient-centered outcomes within real-world healthcare systems.

### 3.2. Breast Cancer

#### 3.2.1. Current AI Landscape and Mature Applications

Breast cancer represents the most advanced AI ecosystem in oncology, a status earned through decades of digitized mammography, standardized biopsy protocols, and institutional investment in screening infrastructure [38]. AI-assisted mammography screening tools have received regulatory approval across multiple jurisdictions, with systems such as Lunit INSIGHT MMG, Transpara (ScreenPoint Medical), and iCAD ProFound AI demonstrating non-inferiority or superiority to radiologist performance in prospective cohorts enrolling tens of thousands of patients [39]. The MASAI trial in Sweden and prospective studies across the UK and US have established that AI-supported reading can maintain diagnostic accuracy while reducing radiologist workload, a finding with immediate healthcare delivery implications given global radiologist shortages [40].

In pathology, AI-based assessment of HER2 status, Ki-67 proliferation index, hormone receptor expression, and lymph node metastasis detection has reached clinical-grade accuracy in multi-institutional studies [41]. The Galen Breast system achieved real-time clinical deployment at Maccabi Healthcare Services, while the Paige Lymph Node tool received FDA Breakthrough Device Designation for lymph node metastasis detection [42]. Quantitative Ki-67 scoring through AI has demonstrated lower inter-observer variability than manual pathologist assessment, directly addressing a longstanding limitation in clinical practice [43]. Deep learning models for morphological-based molecular profiling (MBMP) can now predict estrogen receptor, progesterone receptor, and HER2 status directly from standard H&E images, potentially offering a faster, cheaper alternative to immunohistochemistry in resource-limited settings [44].

**Figure 5:**
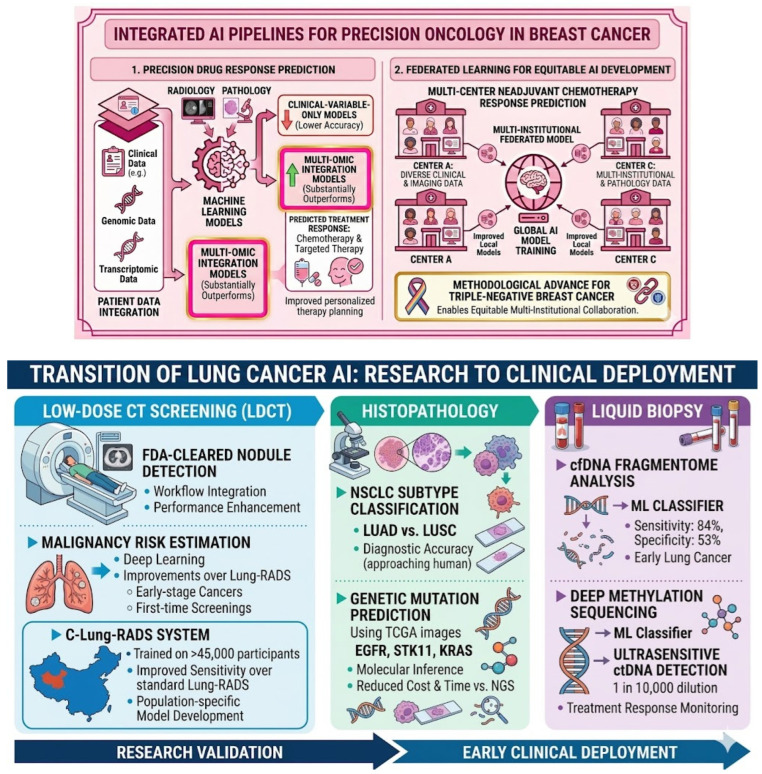
Al-mediated precision oncology in lung and breast cancer [45,46,47,48,49,50,51,52,53,54]. Illustration of Breast-specific (**Top panel**) and lung-specific (**Bottom panel**) Al pipelines integrating radiology, pathology, genomics, and proteomics to improve tumor characterization, treatment selection, and response prediction.

Beyond screening and diagnosis, AI is advancing into surgical planning and drug response prediction [45]. Machine learning models integrating clinical, genomic, and transcriptomic data substantially outperform clinical-variable-only models in predicting chemotherapy and targeted therapy response [46]. Federated learning approaches have enabled multi-center training of models predicting neoadjuvant chemotherapy response in triple-negative breast cancer without requiring raw data sharing, a methodological advance with direct implications for equitable multi-institutional AI development [47] (Figure 5).

#### 3.2.2. Translational Limitations and Remaining Barriers

Despite this maturity, critical translational gaps persist. Post-market surveillance studies consistently reveal performance degradation in dense breast tissue and non-European populations, exposing the gap between controlled validation and real-world deployment [55]. Pivotal clinical trials for AI screening tools enrolled predominantly White participants, with post-market studies documenting disparate false-positive and false-negative rates across skin tone and demographic subgroups [56]. Regulatory frameworks are beginning to mandate subgroup performance reporting, but enforcement mechanisms remain inconsistently applied [57].

The fundamental unresolved question is outcome impact. Breast cancer screening AI achieves high sensitivity in retrospective cohorts [58]. The gap between diagnostic accuracy and clinical benefit is not semantic, previous generations of mammography CAD tools received FDA approval and achieved widespread deployment despite failing to demonstrate mortality benefit in subsequent outcome studies [59]. This history demands that current AI tools be held to a higher evidentiary standard before clinical integration is treated as routine [60]. Additionally, predicting long-term breast cancer risk from AI-analyzed mammograms, while promising through tools like Mirai, requires prospective multi-cohort validation linking AI risk scores to actual cancer development and mortality over clinically meaningful follow-up periods [61].

#### 3.2.3. Future Development Priorities

Priority development areas include algorithmic fairness auditing across breast density categories and ancestral backgrounds, and integration of AI screening outputs with polygenic risk scores and lifestyle-based risk factors for truly personalized screening interval decisions [62]. Priority development areas also include federated learning frameworks that enable multi-national model training without centralizing sensitive patient data [63]. The next transformative step may be AI-augmented liquid biopsy integration, combining circulating tumor DNA methylation profiles with mammographic AI risk scores to create composite early detection tools applicable before radiographic abnormalities appear [64].

### 3.3. Prostate Cancer

#### 3.3.1. Current AI Landscape and Mature Applications

Prostate cancer has benefited from extensive MRI standardization through the PI-RADS reporting framework and widespread adoption of digital pathology, creating conditions favorable for AI development [65]. Multiple AI systems for detecting clinically significant prostate cancer on biparametric MRI have achieved external validation across large independent cohorts [66]. The PI-CAI grand challenge, featuring over 10,000 carefully curated prostate MRI examinations, established performance benchmarks demonstrating that ensemble deep learning models can match or exceed the sensitivity of experienced radiologists while achieving significantly fewer false positives, a finding directly relevant to reducing unnecessary biopsies [67].

In pathology, the PANDA challenge established rigorous benchmarks for AI-based Gleason grading, demonstrating that automated deep learning systems achieve performance concordant with expert uropathologists in independent US and European cohorts [68]. Paige Prostate received FDA approval for biopsy triage in core needle biopsies, representing the first FDA-cleared AI tool for prostate cancer pathology [69]. Explainable AI models that provide visual and textual rationales for detection decisions, incorporating established imaging features like PI-RADS criteria, have shown capacity to improve confidence and reduce reading time for non-expert radiologists, addressing a specific bottleneck in healthcare systems where prostate MRI volumes are growing faster than subspecialty radiologist supply [70].

For treatment planning, fully automated machine learning-generated radiotherapy plans for prostate cancer have demonstrated reproducibility and high performance equivalent to manually generated plans while saving significant planning time, with one prospective study documenting feasibility in a real clinical environment [71]. Multimodal deep learning models integrating histopathology and clinical data from randomized trials have outperformed NCCN risk stratification in predicting long-term outcomes including metastasis and survival, offering a pathway toward therapy personalization [72].

#### 3.3.2. Translational Limitations and Remaining Barriers

Despite strong technical performance, clinical uptake is limited by conceptual uncertainty about overdiagnosis [73]. AI-detected lesions missed by human radiologists may represent clinically insignificant cancers that would have remained on active surveillance indefinitely, and increasing detection of such lesions could paradoxically worsen population-level outcomes through overtreatment [74]. This concern is not academic: the history of PSA-based screening demonstrates that improved detection of prostate cancer does not automatically translate to reduced mortality when a substantial proportion of detected cancers have indolent natural histories [75]. AI tools that improve sensitivity without addressing the clinical significance of detected lesions may recapitulate this harm [76].

Generalizability across imaging systems, acquisition protocols, and healthcare settings remains insufficiently tested [77]. Models trained predominantly in academic European and North American centers may fail when deployed in community hospitals with different MRI field strengths, coil configurations, or patient demographics [78]. Pathology AI for Gleason grading shows excellent mean performance but significant variance in challenging cases, precisely the borderline Gleason 3 + 4 versus 4 + 3 distinctions that most influence treatment decisions and where AI guidance would be most valuable [79].

#### 3.3.3. Future Development Priorities

Future development must address AI-guided focal therapy planning, integrating MRI-detected lesion localization with real-time biopsy guidance to enable targeted treatment of index lesions while sparing surrounding tissue [80]. Integration of PSMA-PET findings with MRI-based AI for biochemical recurrence risk stratification after prostatectomy represents a high-priority translational opportunity [81]. Critically, prospective trials must assess whether AI-guided biopsy decisions reduce overdiagnosis rates while maintaining detection of clinically significant cancers, a study design that requires tracking active surveillance conversion rates and treatment escalation, not merely diagnostic accuracy [82].

### 3.4. Early Adoption: Emerging Clinical Validation (Tier 2)

The cancers in this category possess technical AI development at or near clinical-grade levels, with some regulatory clearances achieved, but face specific barriers, algorithmic bias, generalizability limitations, unclear impact on patient-centered outcomes, or workflow integration resistance, that prevent routine deployment. These malignancies are on a trajectory toward near-clinical status but require targeted evidence generation to bridge identified gaps.

### 3.5. Lung Cancer

#### 3.5.1. Current AI Landscape and Mature Applications

Lung cancer AI has transitioned from research to early clinical deployment across multiple domains [48]. In low-dose CT screening, FDA-cleared nodule detection systems have been integrated into radiology workflows at major health systems, with multiple devices receiving FDA authorization for enhancing workflow efficiency and detection performance [49]. Deep learning models applied to the National Lung Screening Trial dataset demonstrated improvements in malignancy risk estimation over existing Lung-RADS criteria, particularly for early-stage cancers and for patients without prior screening examinations [50]. The C-Lung-RADS system, trained on over 45,000 participants in China, demonstrated substantially improved sensitivity over standard Lung-RADS criteria in internal and external testing cohorts, illustrating both the feasibility of AI-enhanced risk stratification and the importance of population-specific model development [51].

In histopathology, AI has demonstrated capacity to classify non-small cell lung cancer subtypes (LUAD vs. LUSC) from H&E images with performance approaching diagnostic accuracy, and landmark studies have shown that AI trained on TCGA images can predict mutation status of key driver genes including EGFR, STK11, and KRAS, potentially enabling molecular inference from routine slides without the cost and turnaround time of next-generation sequencing [52]. For liquid biopsy, machine learning classifiers applied to cell-free DNA fragmentome analysis have achieved detection sensitivity of 84% with 53% specificity for early lung cancer, a performance level potentially useful for augmenting low-dose CT screening programs [53]. Deep methylation sequencing combined with ML classifiers has demonstrated ultrasensitive circulating tumor DNA detection at dilution ratios as low as 1 in 10,000, offering advantages for treatment response monitoring [54] (Figure 5).

In treatment planning, radiomics is being used to target certain mutations in NSCLC. One mutation that is being targeted is the EGFR mutation. The latest CT radiomics-based model for EGFR mutation prediction achieved an Area under the Receiving Operating Characteristic Curve (AUROC) of 0.80 in external validation. Radiomics has even been able to distinguish the two types of EGFR mutations, 19Del and L858R, with reasonable success. Along with EGFR mutations, CT radiomics has also been able to target ALK mutations in NSCLC when combined with clinical and CT semantic features. This has enabled an AUROC of 0.914. Furthermore, radiomics-based prediction is being used for PD-L1 positivity as well. This model has achieved an AUROC of 0.76 for PD-L1 ≥ 50% [83].

#### 3.5.2. Translational Limitations and Remaining Barriers

The central translational challenge in lung cancer is not detection but risk stratification. Existing AI systems detect more nodules than prior generations of CAD software (VA20/6.4), but clinical benefit depends on accurately distinguishing indolent subcentimeter nodules from early-stage cancers requiring intervention [84]. Overdiagnosis risk is substantial, as demonstrated by the post-NLST literature, and AI systems that increase overall detection rates without improving the positive predictive value for clinically significant malignancies may drive unnecessary invasive procedures [85]. False-positive rates in community-based deployment often exceed those in academic research settings, partly because image acquisition protocols and patient populations differ from training cohorts [86].

Mutation prediction from histology, while technically impressive, has not been validated against the outcome-level question it implicitly claims to address: whetherAI-predicted EGFR mutation status meaningfully guide clinical decisions when molecular testing is available, faster, and already integrated into standard workflows [87]? Until AI histology-based mutation inference is shown to improve outcomes in settings where molecular testing is unavailable or delayed, particularly in low-resource global settings, its clinical role remains supporting rather than replacing established diagnostics [88]. Algorithmic performance also varies significantly by tumor histological subtype, with rare neuroendocrine carcinoma variants representing a recognized weakness of current systems [89].

#### 3.5.3. Future Development Priorities

Priority areas include radiomics-pathology fusion models integrating CT imaging features with histological spatial characteristics for immunotherapy response prediction, a clinically urgent need given the heterogeneity of responses to immune checkpoint inhibition in NSCLC [90]. AI-guided adaptive radiotherapy planning for locally advanced NSCLC, integrating real-time tumor response assessment, represents a high-impact development opportunity [91].

### 3.6. Hepatocellular Carcinoma

#### 3.6.1. Current AI Landscape and Mature Applications

Hepatocellular carcinoma (HCC) benefits from well-established surveillance imaging programs in cirrhotic populations, providing structured datasets that AI systems can exploit [92]. AI models for lesion detection and characterization on ultrasound, CT, and MRI have demonstrated feasibility for automated LI-RADS classification, with deep learning approaches achieving sensitivity and specificity comparable to trained radiologists in retrospective cohorts [93]. Semi-reference-free deconvolution algorithms applied to circulating cell-free DNA methylation profiling have achieved early HCC detection with sensitivity above 86% and specificity approaching 95% in validation cohorts including pre-diagnosis samples, offering a potentially transformative non-invasive detection approach in high-risk patients who are poorly served by standard ultrasound surveillance [94].

AI-based fibrosis staging from liver biopsy whole slide images has demonstrated performance concordant with expert pathologists while reducing inter-observer variability, and computational models integrating imaging, genomic, and clinical features for microvascular invasion prediction, a critical determinant of surgical planning and prognosis, have entered prospective validation in specialized hepatology centers [95]. The clinical urgency of AI in HCC is amplified by the documented underperformance of conventional ultrasound surveillance in detecting early stage lesions in obese patients and those with non-viral liver disease etiologies [96].

AI-based radiomics has helped with predicting TACE treatment in intermediate-stage HCC. Usually, TACE only works for about 60% of patients, but radiomics has enabled the prediction of the initial response to TACE treatment. This AI model has established an AUC of 0.909. AI-based radiomics has also shown promise in predicting response to systemic therapies for HCC [97,98]. Furthermore, these models have been successful at predicting responses to systemic therapies as well. They have predicted HCC patients’ responses to Sorafenib and Lenvatinib [97].

#### 3.6.2. Translational Limitations and Remaining Barriers

Performance degradation in non-viral HCC etiologies represents the most significant translational barrier [99]. Models trained predominantly on hepatitis B and C cohorts, which dominate Asian datasets where HCC prevalence is highest, fail to generalize to Western NASH-related and alcohol-related HCC populations, where tumor morphology, surrounding parenchymal features, and genomic profiles differ substantially [100]. This etiology-specific performance gap means that AI tools validated in high-prevalence East Asian settings may provide little benefit, and potentially misleading reassurance, when deployed in North American or European clinical contexts [101]. External validation across etiology subgroups is largely absent from the published literature [102].

Additionally, HCC imaging diagnosis depends on the LI-RADS standardized lexicon, and AI systems that attempt to predict LI-RADS categories must be validated against the actual clinical consequence of those categories, referral for biopsy, transplant listing criteria, and locoregional therapy eligibility, rather than image-level concordance alone [103]. Rare but clinically important HCC variants (scirrhous HCC, combined HCC-cholangiocarcinoma) represent recognized AI failure modes that require specific attention in model development [104].

#### 3.6.3. Future Development Priorities

Development priorities include etiology-stratified model training and validation, with deliberate oversampling of NASH-related and alcohol-related HCC to address current bias [105]. AI-optimized surveillance interval personalization, moving beyond fixed 6-month ultrasound toward AI-guided risk-adapted protocols incorporating AFP trajectory, Child-Pugh score, and imaging features, represents a high-impact translational opportunity that could reduce both surveillance burden and interval cancer rates simultaneously [106,107].

### 3.7. Melanoma

#### 3.7.1. Current AI Landscape and Mature Applications

Melanoma AI has achieved regulatory milestones with DermaSensor receiving FDA De Novo authorization as the first AI-enabled device for skin cancer detection by primary care physicians in the United States [108]. The pivotal DERM-SUCCESS trial demonstrated 95.5% sensitivity for melanoma and keratinocyte cancers combined, with a negative result by device confirmed by biopsy in 96.6% of cases [109]. Clinical utility studies showed that device use halved missed cancers by primary care physicians, from 18% to 9%, a finding with direct population-level impact given that most skin cancer evaluations occur in primary, not specialty, care settings [110]. AI-based dermoscopy analysis has also demonstrated performance comparable to dermatologists in controlled reader studies across multiple academic centers [111].

In pathology, AI for Breslow depth quantification from whole slide images offers more objective and reproducible staging than manual measurement [112]. Deep learning models for predicting sentinel lymph node status from primary tumor histology are in active development, potentially enabling more precise patient selection for sentinel node biopsy and sparing low-risk patients from an invasive procedure with documented morbidity [113]. AI analysis of tumor-infiltrating lymphocyte density and spatial organization in melanoma pathology slides has shown associations with immunotherapy response, opening a pathway to histology-based immunotherapy biomarker assessment [114].

#### 3.7.2. Translational Limitations and Remaining Barriers

Specificity in real-world primary care settings remains critically low. While sensitivity approaches 95%, specificity in the DermaSensor pivotal trial was only 20.7%, meaning that roughly 80 out of every 100 lesions flagged as requiring specialist attention are actually benign [115]. At population scale, this specificity level would generate an enormous burden of unnecessary dermatology referrals, potentially overwhelming already strained specialist capacity and causing patient anxiety from false-positive results [116]. This specificity gap reflects the fundamental challenge of deploying AI trained on curated lesion-level datasets in general practice settings where the prevalence of true malignancy among presented lesions is much lower [56].

Demographic bias in training data represents a critical equity concern. The DermaSensor post-market surveillance requirement for testing in Fitzpatrick skin types IV–VI reflects the documented imbalance in training datasets, where >95% of participants in pivotal trials were White [117]. Studies have consistently demonstrated performance degradation in darker skin tones, with AI systems showing higher false-negative rates for melanoma in Black and South Asian patients, an algorithmic failure with serious health equity implications given that late-stage melanoma diagnosis is disproportionately common in communities of color [118]. The translational barrier is not algorithmic but social: improving representative data collection requires active community engagement, trust-building with historically excluded populations, and structural changes to research recruitment practices [119].

#### 3.7.3. Future Development Priorities

Future development must prioritize multi-ethnic training cohorts with deliberate representation of Fitzpatrick IV–VI skin types, dermoscopy-pathology fusion models that integrate in vivo imaging with ex vivo histological features for improved specificity, and AI-augmented total body photography platforms for longitudinal high-risk surveillance that can detect lesion change over time, a task where AI has inherent advantages over periodic human examination [120]. Risk stratification models that identify patients who can safely defer sentinel node biopsy, validated against actual nodal status and long-term survival, represent a high-priority development that could meaningfully reduce procedural morbidity [121].

### 3.8. Mixed Translation: Technical Maturity Without Clinical Clarity (Tier 3)

The cancers in this section represent a distinct challenge: AI tools have achieved technical validation and, in some cases, regulatory clearance, but clinical translation remains ambiguous because algorithmic performance has not clearly translated to improved patient outcomes, or because deployment has revealed real-world limitations absent from controlled validation studies.

### 3.9. Colorectal Cancer

#### 3.9.1. Current AI Landscape and Mature Applications

Colorectal cancer exemplifies the disconnect between algorithmic performance and clinical utility [122]. Computer-aided detection (CADe) during colonoscopy has achieved widespread commercial deployment, with multiple FDA-cleared systems (including K211951 and K223473) demonstrating increased adenoma detection rates in randomized controlled trials [123]. The CRCNet deep learning model demonstrated high performance across three independent test cohorts [124]. Multiple companies have achieved FDA clearance or EU certification, and CADx systems for optical polyp diagnosis approach 90% accuracy in controlled settings, achieving negative predictive values sufficient for leave-in situ or resect-and-discard strategies in research environments [125].

In histopathology, AI-based microsatellite instability prediction from H&E images has achieved CE marking through commercial platforms including Owkin’s MSIntuit CRC product, and AI quantification of tumor-stroma ratio from whole slide images has demonstrated prognostic validity for overall survival across independent patient cohorts [126]. Deep learning models for gland instance segmentation in colon histology have demonstrated generalizability across institutions [127]. Prognostic AI models for CRC have been validated using histopathological and multiplex imaging data, with DoMore Diagnostics achieving a CE-marked product predicting prognosis from H&E slides [128]. Prognostic features identified through AI analysis can even be learned and applied by pathologists, a rare example of AI augmenting human pattern recognition rather than replacing it [129].

Both radiomics and DL AI models are being used to treat colorectal cancer currently. These models have shown a very high AUROC of 0.917 in the assessment of lymph node metastasis in rectal and CRC patients. This is a drastic improvement compared to the radiologist assessment, which has an AUROC of 0.688 [130]. Along with radiomics and DL, ML has been used to predict response to chemotherapy. One model has shown a therapeutic response prediction with an overall sensitivity of 92% and specificity of 86%. Furthermore, an ML-based radiomics analysis of the pretreatment CT scan has also been developed. This showed an AUROC of 0.79 [131].

#### 3.9.2. Translational Limitations and Remaining Barriers

Despite these achievements, the clinical utility debate remains unresolved. A meta-analysis of 18,232 patients across 21 randomized trials concluded that CADe increases detection of adenomas but not advanced adenomas, the clinically meaningful endpoint for cancer prevention, while simultaneously increasing the removal rate of nonneoplastic polyps and potentially adding procedure time without benefit [132]. A separate large, randomized trial found that CADe did not improve detection of advanced colorectal neoplasias in a systematic screening program [133]. These findings challenge the assumption that increased adenoma detection automatically translates to reduced colorectal cancer incidence or mortality [134]. The adenoma-carcinoma sequence is well established, but most small adenomas will never progress to cancer within a patient’s lifetime, and their detection and removal generates cost and procedural risk without demonstrable benefit [135].

The clinical adoption of CADx for the resect-and-discard strategy faces similar challenges. A meta-analysis of 7400 diminutive polyps concluded that CADx provided no benefit or harm for resect-and-discard decision-making, questioning its value in routine colonoscopy practice [136]. The gap between research performance and real-world utility reflects the structured nature of validation datasets, which tend to overrepresent clearly neoplastic or clearly benign polyps while underrepresenting the ambiguous intermediate cases where clinical guidance is most needed [137].

#### 3.9.3. Future Development Priorities

Future development must pivot from detection metrics to outcome-driven endpoints. The field needs prospective trials tracking interval cancer rates and colorectal cancer incidence in CADe-assisted versus standard colonoscopy populations over 5–10 year follow-up periods [138]. For pathology AI, the priority is clinical-grade MSI prediction validated against immunotherapy response rates in real-world treatment cohorts, not merely against molecular testing concordance [139]. AI tools for predicting recurrence risk after curative resection, integrating pathological, molecular, and imaging features, represent a high-value development area directly informing adjuvant chemotherapy decisions [140].

### 3.10. Research-Intensive: Technical Feasibility Without Prospective Validation (Tier 4)

The cancers in this section have active AI research programs producing technically impressive results but lack prospective clinical validation demonstrating that AI tools alter clinical decision-making or improve outcomes. These represent the most common state of AI development in oncology, technically mature but translationally premature. The risk is that premature adoption of research-grade tools into clinical practice generates false reassurance or inappropriate clinical decisions.

### 3.11. Brain Tumors (Gliomas)

#### 3.11.1. Current AI Landscape and Mature Applications

Glioma AI has advanced rapidly on the back of standardized MRI protocols and the availability of large annotated datasets through The Cancer Genome Atlas and international glioma consortia [141]. Radiomics models for non-invasive prediction of IDH mutation status and 1p/19q codeletion from preoperative MRI have achieved accuracy approaching 98% in retrospective multi-center cohorts, using deep CNN architectures that capture spatial and textural features invisible to visual inspection [142]. These molecular inference capabilities are clinically significant because IDH status fundamentally determines glioma diagnosis, prognosis, and treatment planning under the 2021 WHO classification [143]. Deep learning for glioma grade classification from multiparametric MRI achieves sensitivity above 95% in retrospective testing [144].

Intraoperative AI represents perhaps the most striking translational advance in brain tumor care. The Sturgeon system, which uses rapid nanopore sequencing combined with neural networks to subclassify CNS tumors during surgery from sparse methylation profiles, achieves concordance exceeding 95% with reference standard DNA methylation profiling while operating within surgical time constraints [145]. Similarly, DEPLOY classifies CNS tumors into 10 major categories from histopathology within a clinically relevant short time frame [146]. These tools address a genuine unmet need: real-time molecular information during craniotomy that could influence surgical extent decisions and inform immediate postoperative management [147].

For treatment planning and surveillance, radiomics models predicting gamma knife radiosurgery response for brain metastases and distinguishing pseudoprogression from true tumor progression after radiotherapy have demonstrated feasibility in retrospective studies [148]. AI models integrating preoperative MRI imaging markers accurately predict patient survival and classify GBM molecular subtypes, identifying distinctive radiographic phenotypes correlating with clinical outcomes [149].

#### 3.11.2. Translational Limitations and Remaining Barriers

The critical translational gap is prospective outcome validation. No studies have demonstrated that AI-predicted molecular status alters surgical planning in ways that improve patient outcomes compared to waiting for standard neuropathology reports [150]. Neurosurgeons typically resect tumors regardless of suspected molecular subtype, the extent of safe resection is determined by functional anatomy, not molecular classification, raising the question of when intraoperative molecular AI predictions would actually change clinical decisions [151]. This does not invalidate technology but underscores the need for carefully designed prospective trials that pre-specify the clinical decisions to be influenced by AI predictions and measure patient-centered outcomes downstream [152].

Rare glioma subtypes, particularly high-grade astrocytoma with piloid features, introduced in the 2021 WHO classification, require methylome profiling for accurate diagnosis and represent a recognized failure mode for imaging-based AI systems trained predominantly on common subtypes [153]. The 2021 WHO reclassification of CNS tumors fundamentally reorganized the diagnostic landscape, and AI systems trained on pre-2021 diagnostic categories may produce systematically incorrect outputs when applied to contemporary cases classified under the revised framework [154]. This represents a broader challenge: AI models trained on historical datasets are vulnerable to conceptual drift as clinical diagnostic criteria evolve [155].

#### 3.11.3. Future Development Priorities

Development priorities include AI-guided surgical margin delineation integrating real-time MRI with intraoperative ultrasound and fluorescence imaging, pseudoprogression versus true progression differentiation integrating serial MRI radiomics with clinical trajectory and treatment history, and longitudinal liquid biopsy AI for non-invasive monitoring of tumor evolution and treatment response [156]. Most critically, the field requires prospective trials in which AI-predicted molecular status is used to guide surgical decision-making, in clearly defined scenarios such as extent of resection in suspected IDH-mutant versus IDH-wildtype tumors, with outcome tracking over clinically meaningful follow-up periods [157].

### 3.12. Pancreatic Ductal Adenocarcinoma

#### 3.12.1. Current AI Landscape and Mature Applications

Pancreatic ductal adenocarcinoma (PDAC) represents one of the most compelling cases for AI development given its catastrophically poor prognosis, rooted in late-stage diagnosis in the vast majority of patients [158]. AI models predicting pancreatic cancer risk from electronic health records have achieved validation in large retrospective cohorts of 500,000+ patients by identifying subtle pre-diagnostic patterns in routine laboratory results, medication use, and clinical encounter trajectories [159]. Deep learning applied to disease trajectory data has demonstrated capacity to identify high-risk individuals up to 3 years before clinical diagnosis [160]. NCI-supported researchers have harnessed deep learning trained on population-scale disease data to predict individual pancreatic cancer risk, laying groundwork for earlier detection [161].

In imaging, AI-based automated segmentation of the pancreas and pancreatic lesions from CT and MRI has achieved accuracy approaching manual radiologist delineation, enabling more consistent volumetric assessment of lesion growth in individuals with pancreatic cysts under surveillance [162]. Radiomics models for distinguishing pancreatic ductal adenocarcinoma from autoimmune pancreatitis, a clinically important differential diagnosis that significantly affects management, have demonstrated feasibility in multi-center cohorts [163]. For resectability assessment, AI integration of vascular contact measurements with radiological staging has shown promise in standardizing a subjective judgment that significantly impacts surgical referral decisions [164].

#### 3.12.2. Translational Limitations and Remaining Barriers

Despite high clinical need, PDAC AI translation faces fundamental barriers. The most critical is the absence of a validated early detection strategy for AI to augment [165]. Unlike breast or lung cancer, where screening programs identify asymptomatic individuals who can be triaged by AI, no population-level pancreatic cancer screening exists [166]. AI risk models applied to primary care EHR data could in principle identify high-risk individuals warranting surveillance, but what surveillance [167]? Endoscopic ultrasound and MRI can detect early pancreatic lesions but lack specificity, and there is no evidence that detecting early PDAC through EUS-based surveillance improves mortality [168]. AI risk scores without actionable surveillance pathways produce clinical anxiety without benefit [169].

Data scarcity is a structural problem. Pancreatic cancer affects approximately 0.5% of the population over a lifetime, and even large academic centers accumulate limited resected specimen cohorts for training pathology or surgical planning models [170]. Multi-institutional federated data sharing is essential but complicated by proprietary institutional interests and regulatory constraints [171]. Radiomics models for predicting margin-positive resection or nodal involvement have been published in retrospective single-institution cohorts but consistently fail to validate externally, a pattern reflecting overfitting to institution-specific surgical technique and imaging protocol variability [172].

#### 3.12.3. Future Development Priorities

The highest priority is building the clinical infrastructure for AI-guided PDAC early detection: prospective studies embedding AI risk stratification in primary care workflows, linked to defined surveillance pathways (EUS, MRI, multi-analyte blood tests), with pre-specified clinical decision rules and outcome tracking [160]. AI-guided biomarker discovery from multi-analyte liquid biopsy panels, combining ctDNA, CA19-9 trajectory, microRNA, and metabolomics, represents a technically promising approach requiring validation in prospectively collected pre-diagnosis biobanks [173]. For surgical planning, AI integration with real-time intraoperative imaging to predict resectability and guide vascular dissection decisions offers a high-impact near-term opportunity [174].

### 3.13. Ovarian Cancer

#### 3.13.1. Current AI Landscape and Mature Applications

Ovarian cancer AI research has concentrated on three domains: imaging-based mass triage, pathological subtype classification, and molecular biomarker prediction [175]. AI-augmented ultrasound interpretation for adnexal mass triage has demonstrated feasibility in distinguishing benign from malignant masses, with deep learning models trained on longitudinal imaging datasets showing improved sensitivity over the established O-RADS reporting system in preliminary retrospective analyses [176]. In pathology, AI for histological subtype classification, discriminating high-grade serous, clear cell, endometrioid, and mucinous ovarian carcinomas, has demonstrated performance comparable to subspecialty gynecological pathologists in multi-institutional studies, relevant because subtype determination increasingly drives treatment selection in the era of PARP inhibitors [177].

Homologous recombination deficiency (HRD) prediction from histological features represents an emerging and clinically significant application [178]. HRD status determines eligibility for PARP inhibitor maintenance therapy in platinum-sensitive ovarian cancer, yet genomic HRD testing has variable availability and turnaround times across healthcare settings [179]. AI models predicting HRD status from H&E whole slide images have shown initial feasibility, offering a potential pathway to equitable access to biomarker-guided therapy [180]. Multi-omics AI integrating genomics, transcriptomics, and clinical features for predicting platinum sensitivity and overall survival has shown discriminative capacity in retrospective cohort studies [181].

#### 3.13.2. Translational Limitations and Remaining Barriers

Translational barriers in ovarian cancer are multi-layered. The absence of a validated early detection paradigm, the fundamental challenge that has resisted decades of biomarker and imaging research, limits the impact of AI detection tools, because AI cannot improve upon a screening strategy that does not exist [182]. The UKCTOCS trial, the largest ovarian cancer screening randomized trial ever conducted, failed to demonstrate mortality benefit from CA-125-based or ultrasound-based screening despite detecting more early-stage cancers, a sobering reminder that detection without an effective intervention pathway does not translate to survival benefit [183].

Histological heterogeneity within high-grade serous ovarian carcinoma, the dominant subtype, complicates AI pathology model development, because regional sampling variation within a single tumor can produce divergent histological patterns in the same patient [184]. AI models trained on single-biopsy datasets may fail when applied to resection specimens exhibiting spatial heterogeneity [185]. Additionally, multi-omics AI models face validation challenges due to the rarity of prospectively collected, biobank-quality multi-modal datasets with long-term outcome annotation in ovarian cancer [186].

#### 3.13.3. Future Development Priorities

Priority development areas include AI-guided composite early detection combining transvaginal ultrasound, CA-125 kinetics, and multi-analyte blood-based biomarkers in a unified risk model validated in high-risk populations (BRCA1/2 carriers, Lynch syndrome) [187]. AI-based HRD prediction from histology requires prospective validation against PARP inhibitor response rates in clinical trial contexts, not merely against genomic testing concordance, before it can be considered a validated biomarker [188]. Spatial multi-omics AI that integrates tumor-immune microenvironment mapping with clinical outcome data represents a longer-term development priority addressing the recognized prognostic importance of tumor-infiltrating lymphocytes and immune checkpoint expression in ovarian cancer [189].

### 3.14. Pilot Phase: Focused Proof-of-Concept with Limited Generalizability (Tier 5)

Cancers in this category have demonstrated AI feasibility in specific institutional or geographic contexts but face severe generalizability constraints rooted in disease heterogeneity, geographic variation in disease biology, or population-specific imaging practice differences. Promising pilot results have not been translated to multi-institutional validation, and claims of clinical readiness are premature.

### 3.15. Gastric Cancer

#### 3.15.1. Current AI Landscape and Mature Applications

Gastric cancer AI has advanced most rapidly in endoscopic detection, driven by large, digitized endoscopy archives in high-prevalence East Asian populations [190]. Real-time AI systems for detecting early gastric cancers and precancerous lesions during endoscopy, including gastric intestinal metaplasia, dysplasia, and early mucosal cancers, have demonstrated sensitivity and specificity approaching expert endoscopist performance in prospective single-center Chinese and Japanese cohorts [191]. These systems operate in real-time during endoscopy, providing augmented detection for endoscopists who examine a high volume of patients where fatigue and attention lapses are known to cause missed lesions [192].

In pathology, AI for Lauren classification (intestinal vs. diffuse type) from whole slide images has shown feasibility in retrospective studies, and HER2 scoring automation for gastric adenocarcinoma, where HER2-positive status determines eligibility for trastuzumab, offers a standardization benefit comparable to its breast cancer application [193]. AI-based prediction of response to perioperative chemotherapy (FLOT regimen) from pretreatment CT imaging and biopsy pathology has entered exploratory investigation, driven by the clinical urgency of identifying non-responders before surgery [194].

#### 3.15.2. Translational Limitations and Remaining Barriers

External validation outside high-incidence East Asian settings is virtually absent. Models trained on Japanese and Chinese endoscopy cohorts, where early gastric cancer constitutes a larger proportion of detected disease due to organized mass screening programs, fail when deployed in Western settings where gastric cancer is typically detected at advanced stages and disease distribution within the stomach differs [195]. This external validation gap reflects not merely a technical limitation but a deeper biological reality: gastric cancer in Japan is predominantly intestinal type arising on a background of H. pylori-associated atrophic gastritis, while Western cases include a higher proportion of diffuse-type gastric cancers with fundamentally different morphological features and carcinogenesis pathways. AI models trained in one biological context cannot be assumed to generalize to the other [196].

#### 3.15.3. Future Development Priorities

Future work requires multi-regional validation cohorts spanning East Asian, Western European, and South American populations, regions with distinct gastric cancer epidemiology and disease biology [197]. AI tools for predicting perioperative chemotherapy response using integrated CT-pathology models require prospective validation in randomized trial contexts before clinical deployment [198]. Federated learning frameworks enabling multi-national model training without data centralization are particularly important for gastric cancer AI, given the geographic concentration of high-quality training data in East Asia and the need for models that perform across diverse patient populations [63].

### 3.16. Esophageal Cancer

#### 3.16.1. Current AI Landscape and Mature Applications

Esophageal cancer AI has focused primarily on Barrett’s esophagus surveillance, the precancerous condition leading to esophageal adenocarcinoma, and on endoscopic detection of squamous dysplasia in high-risk populations [199]. AI systems for real-time endoscopic identification of dysplastic Barrett’s segments have demonstrated sensitivity improvements over standard white-light endoscopy in prospective pilot studies, with the added clinical value of targeting biopsies to areas of highest malignant potential, potentially reducing the number of random biopsies required per surveillance procedure [200]. Automated detection of subtle mucosal abnormalities in squamous cell carcinoma surveillance is under investigation in high-incidence populations in China and Iran [201].

For staging and treatment planning, AI-based analysis of endoscopic ultrasound images for T-staging of esophageal tumors and lymph node involvement assessment has shown preliminary feasibility, addressing a critical staging decision point that determines eligibility for neoadjuvant therapy [202]. Radiomics features from pretreatment CT and PET imaging have been investigated for predicting pathological complete response to neoadjuvant chemoradiation, the key outcome determination that guides surgical planning in locally advanced disease [203].

#### 3.16.2. Translational Limitations and Remaining Barriers

Surveillance outcome validation is the defining translational challenge. Demonstrating that AI-targeted Barrett’s surveillance biopsies detect dysplasia at rates non-inferior to random biopsy protocols requires large prospective studies tracking dysplasia detection rates, cancer development, and procedure efficiency across multiple endoscopy centers with varying operator expertise [204]. The fundamental clinical question, whether AI-augmented Barrett’s surveillance reduce esophageal adenocarcinoma mortality, requires decade-long follow-up in randomized trials that have not yet been designed, let alone completed [205].

#### 3.16.3. Future Development Priorities

Prospective outcome trials for AI-augmented Barrett’s surveillance are the top priority, specifically designed to assess whether AI-targeted biopsy strategies can reduce random biopsy burden without sacrificing dysplasia detection rates [206]. AI-guided adaptive radiotherapy planning for esophageal cancer, integrating mid-treatment PET response assessment with dose escalation or de-escalation decisions, represents a high-clinical-impact development opportunity [207]. Integration with multi-analyte liquid biopsy for non-invasive disease monitoring after definitive chemoradiation, avoiding esophageal intubation in frail patients, represents a patient-centered innovation deserving investigation [208].

### 3.17. Cervical Cancer

#### 3.17.1. Current AI Landscape and Mature Applications

Cervical cancer AI occupies a unique position: its greatest translational opportunity lies in low- and middle-income countries (LMICs) where the burden of disease is highest yet access to trained cytologists, colposcopists, and HPV testing infrastructure is most limited [209]. AI-augmented visual inspection with acetic acid (VIA) using smartphone-based algorithms has demonstrated sensitivity approaching cytology-based screening in prospective studies in sub-Saharan Africa and South Asia, offering a potentially transformative screening tool deployable without laboratory infrastructure [210]. Automated cervical cytology analysis using deep learning has achieved performance approaching expert cytopathologist accuracy in LBC slide classification, with potential to address the severe shortage of trained cytologists in high-burden settings [211].

In colposcopy, AI-guided image analysis for identifying acetowhite regions requiring biopsy has demonstrated improved sensitivity for high-grade cervical intraepithelial neoplasia, particularly for identifying the squamocolumnar junction in challenging cases [212]. AI for p16/Ki-67 dual staining interpretation offers potential standardization of a slide-level triage test that has variable inter-observer agreement when assessed manually. Integration with HPV genotyping data for risk-adapted colposcopy referral decisions is under active investigation [213]. As far as treatment, AI models have started to be used in high-dose-rate brachytherapy (HDR-BT) for cervical cancer, but it is still in its initial stages [214,215].

#### 3.17.2. Translational Limitations and Remaining Barriers

Generalizability across global populations is the central translational challenge. AI models trained on high-resource Western screening cohorts fail in LMIC deployment due to differences in HPV genotype distribution (HPV 45, 31, and 33 more prevalent in Africa versus HPV 16/18 dominance in European training data), disease prevalence affecting positive predictive value, image acquisition quality from low-cost smartphone cameras versus high-resolution laboratory scanners, and underlying cervical ecology differences linked to HIV prevalence in high-burden regions [216]. Performance for glandular lesions (endocervical adenocarcinoma precursors) is consistently lower than for squamous lesions across all AI cervical screening tools, reflecting both the relative rarity of these lesions in training datasets and their distinct morphological features [217].

#### 3.17.3. Future Development Priorities

Development must prioritize LMIC-specific model training using locally collected data from high-burden settings, with validation against clinical outcomes (CIN2+ detection, cancer incidence) rather than cytology concordance [218]. Regulatory pathways in most LMICs lack frameworks for AI-based medical device approval, creating a fundamental barrier to deployment even when tools demonstrate technical feasibility [219]. Advocacy for LMIC-inclusive regulatory frameworks and WHO prequalification of AI screening tools is as important as algorithmic development [220]. AI for HPV genotyping integration, enabling AI-predicted extended genotype risk scores from liquid-based cytology specimens without separate molecular testing, represents a potentially impactful development for resource-limited settings [221].

### 3.18. Limited Translation: Structural Barriers to AI Development

The cancers in this section face structural barriers to AI development that cannot be overcome through algorithmic refinement alone. These barriers include extreme biological heterogeneity within diagnostic categories (sarcoma), fundamental ethical and regulatory constraints on data collection (pediatric oncology), workflow incompatibility between AI architectures and established diagnostic modalities (hematologic malignancies), and the compounding effects of low incidence on sample size adequacy. Meaningful AI translation in these domains requires coordinated policy, data sharing, and regulatory intervention alongside technical development.

### 3.19. Hematologic Malignancies (Leukemia and Lymphoma)

#### 3.19.1. Current AI Landscape and Mature Applications

Hematologic malignancy AI has advanced most notably in morphological analysis of peripheral blood smears and bone marrow aspirates [222]. Deep learning models for automated blast cell counting in acute myeloid leukemia (AML) bone marrow samples have demonstrated accuracy approaching manual differential counts, with the advantage of processing hundreds of cells per second without fatigue-related performance degradation [223]. AI for lymphocyte subtype classification in chronic lymphocytic leukemia from peripheral blood has shown preliminary feasibility [224]. In digital pathology, AI for classifying diffuse large B-cell lymphoma subtypes, germinal center B-cell versus activated B-cell phenotype, from H&E images has been investigated as a surrogate for gene expression profiling, with mixed results that reflect the biological complexity of molecular subtyping from morphology alone [225].

Single-cell AI applied to flow cytometry data represents a technically mature domain with direct clinical workflow applications [226]. Machine learning classifiers for minimal residual disease (MRD) detection in acute lymphoblastic leukemia, identifying rare leukemic blasts among millions of normal hematopoietic cells, have demonstrated sensitivity comparable to gold-standard next-generation sequencing-based MRD assays while using existing flow cytometry infrastructure [227]. AI for predicting response to hypomethylating agents in myelodysplastic syndrome from bone marrow biopsy features has entered exploratory investigation [228].

#### 3.19.2. Translational Limitations and Remaining Barriers

The fundamental barrier to hematologic malignancy AI translation is workflow incompatibility. Clinical hematology diagnosis depends on multiparametric flow cytometry providing immunophenotypic data across dozens of markers simultaneously, a data type that image-based AI architectures cannot replicate from morphology alone [229]. Hematopathologists integrate morphology, immunohistochemistry, flow cytometry, cytogenetics, and molecular genetic testing in a hierarchical diagnostic framework where each modality plays a defined role [230]. AI tools that improve one component (morphological blast counting) without integrating the full diagnostic framework provide incremental utility at best and may generate false confidence if their outputs are interpreted outside clinical context [231].

Biological complexity presents additional barriers. Acute myeloid leukemia encompasses dozens of cytogenetically and molecularly defined subtypes with distinct prognoses and treatment responses [232]. AI models trained on mixed AML cohorts may achieve acceptable aggregate performance while performing poorly for specific subtypes, particularly rare but clinically important entities like core-binding factor AML or therapy-related AML, where training sample sizes are insufficient for robust subtype-specific learning [233]. Dynamic disease biology in treated patients, where leukemic clone composition shifts in response to therapy, means that AI models trained on pre-treatment morphology may not generalize to post-treatment assessment contexts [234].

#### 3.19.3. Future Development Priorities

Priority development areas include integrated multimodal AI combining flow cytometry immunophenotyping, morphological analysis, and molecular genetic features for unified diagnostic and risk stratification output, a tool that would genuinely augment, rather than partially replicate, the hematopathologist diagnostic workflow [235]. AI-guided clonal architecture analysis from single-cell sequencing data, tracking sub-clone evolution under therapeutic pressure in AML and CLL, represents a scientifically significant and clinically actionable application requiring validation against treatment response and survival endpoints [236].

### 3.20. Head and Neck Squamous Cell Carcinoma

#### 3.20.1. Current AI Landscape and Mature Applications

Head and neck squamous cell carcinoma (HNSCC) AI research has addressed a disease with striking biological heterogeneity: HPV-positive oropharyngeal cancers behave almost as a distinct disease from HPV-negative tumors, with dramatically different prognoses and emerging treatment de-escalation strategies [237]. AI for HPV status prediction from H&E histology has demonstrated feasibility, offering a potential non-molecular surrogate for p16 immunohistochemistry in resource-limited settings [238]. Radiomics features extracted from pretreatment CT and PET imaging have been investigated for predicting locoregional control after definitive chemoradiation, with models integrating primary tumor volume, texture features, and nodal burden showing discriminative capacity in retrospective studies [239].

AI-guided adaptive radiotherapy planning represents a clinically urgent application. Head and neck tumors often undergo significant volume change during the 6–7 week course of radiotherapy, as primary tumor and involved lymph nodes respond to treatment, and adaptive replanning to account for these anatomical changes can reduce unnecessary irradiation of normal tissues including salivary glands, spinal cord, and swallowing musculature [206]. Automated re-segmentation of tumors and organs-at-risk on weekly CT images during treatment is technically feasible with deep learning, and several prospective studies are evaluating whether AI-guided adaptive replanning reduces xerostomia and dysphagia, the major long-term toxicities that impair quality of life in HNSCC survivors [240].

#### 3.20.2. Translational Limitations and Remaining Barriers

Small, heterogeneous datasets are the dominant barrier. HNSCC spans multiple anatomical subsites (oropharynx, larynx, oral cavity, hypopharynx, nasopharynx, salivary glands) with distinct etiologies, treatment approaches, and prognostic determinants [241]. AI models trained on mixed HNSCC cohorts without stratifying by subsite and HPV status learn confounded associations that fail to generalize to subsite-specific clinical questions [242]. Multi-institutional datasets with granular subsite, HPV status, treatment, and outcome annotation are required but rarely assembled due to institutional data governance constraints [243]. Imaging protocol heterogeneity, different CT slice thicknesses, MRI sequences, and PET tracers across institutions, further limits radiomics model generalizability [244].

#### 3.20.3. Future Development Priorities

AI-guided treatment de-escalation decision support for HPV-positive oropharyngeal cancer, identifying patients who can safely undergo reduced-dose or reduced-field radiotherapy without compromising oncological outcomes, represents the highest-impact near-term translational opportunity [245]. This requires large multi-institutional prospectively annotated datasets stratified by HPV status, p16 expression, and treatment approach [246]. AI-augmented ctDNA surveillance for post-treatment disease monitoring offers a minimally invasive alternative to imaging-based follow-up that could detect molecular recurrence months before radiographic evidence, enabling earlier salvage intervention [247].

### 3.21. Bladder Cancer

#### 3.21.1. Current AI Landscape and Mature Applications

Bladder cancer AI has focused primarily on two domains: cystoscopy augmentation and pathological staging accuracy [248]. AI-assisted cystoscopy for flat lesion detection, particularly for carcinoma in situ, which is notoriously difficult to visualize, has entered feasibility testing with computer vision systems trained on blue-light and white-light cystoscopy video [249]. Automated detection of papillary lesions during cystoscopy, including small recurrent tumors that may be missed during rapid surveillance procedures, has demonstrated sensitivity improvements in pilot studies conducted at single academic centers [250].

In pathology, AI for distinguishing non-muscle-invasive from muscle-invasive bladder cancer from TURBT whole slide images, the critical staging decision determining whether radical cystectomy is required, has shown initial feasibility in retrospective single-institution cohorts [251]. Automated quantification of muscularis propria in TURBT specimens, a technical challenge that affects staging accuracy when muscle is absent from specimens, has been proposed as an AI quality assurance application [252]. For urine cytology, AI-based automated slide analysis for detecting malignant urothelial cells has been investigated as a standardization tool for the Paris System for Reporting Urinary Cytology [253].

Radiomics has been used to predict chemotherapy response in bladder cancer. In particular, 91 different radiomic features have been applied to CT assessments of bladder cancer, achieving an AUC of 0.77. Furthermore, a CNN model has been utilized using pre- and post-CT data. This model achieved an AUC of 0.79 [254,255].

#### 3.21.2. Translational Limitations and Remaining Barriers

Workflow integration in a disease managed through established urological surveillance protocols is the primary translational barrier [256]. Bladder cancer surveillance cystoscopy follows defined intervals (every 3–6 months initially, extending with negative results) embedded in institutional scheduling systems [257]. AI tools that augment cystoscopy must integrate into existing endoscopy workflows without adding procedure time or requiring additional equipment setup [258]. The value proposition must be clearly demonstrated: how many additional clinically significant recurrences are detected per 1000 surveillance procedures, and what is the false-positive cost in additional biopsy procedures and patient anxiety [259]?

#### 3.21.3. Future Development Priorities

Urine-based liquid biopsy AI, using cell-free DNA methylation patterns, urine cytology automation, or multi-analyte biomarker panels, for non-invasive recurrence monitoring between surveillance cystoscopy intervals represents the highest-impact development priority [260]. If AI-based urine testing could safely extend surveillance cystoscopy intervals in low-risk non-muscle-invasive bladder cancer patients, the patient experience and healthcare cost benefits would be substantial [261]. Validation against cystoscopy-confirmed recurrence rates in prospective cohorts is essential before clinical recommendation [262]. AI integration with narrow-band imaging and photodynamic diagnostics during cystoscopy for carcinoma in situ detection requires prospective multi-center validation [263].

### 3.22. Endometrial Cancer

#### 3.22.1. Current AI Landscape and Mature Applications

Endometrial cancer has been underrepresented in AI research relative to its rising incidence, but emerging work addresses clinically critical decision points [264]. The TransCAN/PORTEC molecular classification framework, stratifying endometrial cancers into POLE ultramutated, mismatch repair deficient, p53 abnormal, and no specific molecular profile subgroups, has transformed treatment planning and created a specific need for AI tools that can predict molecular subgroup membership from histological or imaging features without requiring full molecular profiling [265]. Preliminary AI models for predicting POLE mutant status and MMR deficiency from H&E images have shown feasibility in retrospective cohorts, with the clinical value proposition of enabling molecular subgroup-informed treatment decisions in resource-limited settings lacking access to molecular testing [266].

Radiomics for predicting lymph node metastasis risk from preoperative MRI, the critical staging determination that drives decisions about sentinel node biopsy or systematic lymphadenectomy, has demonstrated discriminative capacity in retrospective multi-center studies, with models integrating MRI texture features and tumor volume showing improved accuracy over clinical risk scores alone [267]. These tools could support surgical de-escalation in low-risk patients by identifying individuals unlikely to harbor nodal disease, sparing them from lymphadenectomy-associated morbidity including lymphedema [268].

#### 3.22.2. Translational Limitations and Remaining Barriers

Prospective data scarcity is the central barrier. Most endometrial cancer AI development relies on TCGA retrospective cohorts with limited clinical annotation, or on single-institutional pathology archives not linked to treatment and outcome data [269]. The TCGA endometrial cancer cohort, while valuable for discovery, contains patients enrolled before the molecular classification era, limiting relevance to contemporary clinical questions [270]. Importantly, endometrial cancer AI for molecular subgroup prediction must be validated not against molecular testing concordance alone but against the clinical outcome question: does AI-predicted molecular subgroup status guide adjuvant therapy decisions that improve disease-free or overall survival [271]? This requires linking AI predictions to treatment decisions in prospectively enrolled cohorts with long-term follow-up [272].

#### 3.22.3. Future Development Priorities

Prospective validation of AI-based molecular subgroup prediction in treatment-naive endometrial cancer patients, linked to adjuvant therapy decisions and survival outcomes, is the top priority [266]. AI integration with minimally invasive biopsy specimens, enabling molecular classification from office-based endometrial sampling rather than surgical resection, would expand access to molecular-guided treatment planning [273]. AI-guided decision support for sentinel node biopsy versus systematic lymphadenectomy, integrating MRI radiomics with clinical and histological risk factors, requires randomized validation against lymphedema rates and nodal staging accuracy [274].

#### 3.22.4. Very Limited Translation: Rare, Heterogeneous, and Data-Sparse Cancers

The final group of cancers faces translational barriers so fundamentally that conventional AI development approaches are insufficient. Sample size constraints from disease rarity, extreme biological heterogeneity within diagnostic categories, unique ethical frameworks governing research in vulnerable populations, and near-total absence of organized data-sharing infrastructure collectively limit AI translation. Progress in these domains requires coordinated international data governance frameworks and methodological innovation, including federated learning, few-shot learning, and synthetic data augmentation, rather than incremental algorithmic improvement.

### 3.23. Sarcoma

#### 3.23.1. Current AI Landscape and Mature Applications

Sarcoma represents the most data-sparse oncology domain in AI research, with over 70 distinct histological subtypes combined affecting fewer than 15,000 new patients annually in the United States [275]. AI research in sarcoma has concentrated on two areas: radiomics for treatment response assessment and histopathological subtype classification [276]. Radiomics models extracting texture and morphological features from MRI of extremity soft tissue sarcomas have demonstrated preliminary feasibility for predicting histological response to neoadjuvant chemotherapy, a clinically significant endpoint that guides limb-sparing surgery planning [277]. In pathology, AI systems for distinguishing atypical lipomatous tumor from well-differentiated liposarcoma, a diagnostically challenging distinction with major management implications, have shown single-institution feasibility using MDM2 amplification pattern analysis from H&E images [278].

AI integration with genomic profiling data for fusion gene detection, which defines diagnostic categories and targeted therapy eligibility in synovial sarcoma (SS18::SSX), Ewing sarcoma (EWSR1::FLI1), and alveolar rhabdomyosarcoma (PAX::FOXO1), represents a nascent but potentially impactful application [279]. Deep learning models that can predict fusion gene status from H&E morphology could prioritize specimens for targeted molecular testing, reducing turnaround time in diagnostically ambiguous cases [280].

#### 3.23.2. Translational Limitations and Remaining Barriers

Extreme rarity is the dominant barrier. Even pooling sarcoma cases across major academic referral centers over a decade yields cohorts insufficient for training robust deep learning models for rare subtypes [281]. The largest published sarcoma pathology AI datasets contain a few hundred cases of any single subtype, sample sizes that would be considered inadequate for training reliable models in common cancers [282]. This rarity is compounded by biological heterogeneity: high-grade soft tissue sarcomas exhibit such diverse histological appearances that models trained on one subtype do not generalize to others, and mixed training datasets produce heterogeneous models that are adequate for no specific diagnosis [283].

Ground-truth annotation in sarcoma pathology is additionally challenging because diagnostic concordance among expert pathologists is lower for sarcoma than virtually any other solid tumor type [284]. AI models trained on labels from a single institutional pathologist may learn subtype-specific labeling conventions that differ from other expert pathologists, reducing generalizability even without changing the patient population [285].

#### 3.23.3. Future Development Priorities

International consortium-based data sharing through organizations, including the Connective Tissue Oncology Society (CTOS) and Bone Tumor Reference Centre networks, enabling federated learning across European, North American, and Asian sarcoma centers, is the essential enabling step for meaningful AI development [286]. Few-shot learning approaches, designed to achieve reasonable performance from small, labeled datasets by leveraging pre-trained feature representations from common tumors, require investigation as a potential pathway to sarcoma AI development within realistic sample size constraints [287]. AI-guided digital pathology consultation tools, connecting community pathologists with rare sarcoma diagnoses to expert reference centers through standardized WSI platforms, represent a near-term implementable application that does not require large training datasets [288].

### 3.24. Pediatric Solid Tumors

#### 3.24.1. Current AI Landscape and Mature Applications

Pediatric solid tumor AI faces a distinctive combination of biological, ethical, and logistical challenges that fundamentally distinguish it from adult oncology AI development [289]. Neuroblastoma risk stratification, the most clinically studied pediatric solid tumor AI application, has shown preliminary promise using radiomics features from CT and MIBG imaging to augment INRG risk classification, potentially improving identification of ultra-high-risk patients who benefit from intensified therapy [290]. In pathology, AI for histological classification of nephroblastoma (Wilms tumor) histological subtypes, distinguishing favorable from diffuse anaplastic histology, has demonstrated feasibility in retrospective institutional cohorts, with relevance to chemotherapy intensity decisions [291].

For Ewing sarcoma, a rare pediatric bone and soft tissue cancer with characteristic EWSR1 fusions, AI for distinguishing Ewing sarcoma from morphologically similar small round blue cell tumors using H&E features has been investigated as a triage tool to guide molecular testing [292]. Genomic AI for identifying targetable alterations in pediatric tumors, particularly ALK amplification in neuroblastoma and NTRK fusions across pediatric solid tumor types, from whole genome sequencing data has advanced alongside adult precision oncology platforms, with pediatric cohorts incorporated into multi-cancer genomic profiling programs [293].

#### 3.24.2. Translational Limitations and Remaining Barriers

Ethical and regulatory constraints on pediatric data collection represent the most distinctive barrier [294]. Parental consent requirements, heightened data protection frameworks applicable to minors, and institutional review board restrictions on secondary data use collectively reduce the data volumes available for AI training [295]. Even when pediatric cancer data are nominally available, re-identification risks in small patient populations, where rare tumor diagnoses combined with geographic and demographic features can uniquely identify individuals, impose conservative data sharing restrictions that prevent the large-scale federated learning approaches envisioned for adult oncology [296].

Biological distinctions between pediatric and adult cancer invalidate the assumption that adult-trained models can be applied to pediatric contexts [297]. Pediatric tumors arise from developmental lineages, exhibit distinct genomic landscapes (with lower mutational burden but distinct structural variants), and present in growing organs with different normal tissue references [298]. Models trained on adult imaging, even for ostensibly similar tumor types, may fail catastrophically in pediatric contexts due to organ size differences, developmental tissue characteristics, and age-dependent disease prevalence patterns [299].

#### 3.24.3. Future Development Priorities

International pediatric oncology consortia, Children’s Oncology Group (COG) in North America, SIOP Europe, and ITCC, must lead data harmonization and federated learning framework development as the essential enabling infrastructure for pediatric AI [300]. Harmonized imaging protocols, standardized pathology annotation frameworks, and shared genomic data platforms with appropriate consent frameworks for secondary AI research represent the foundational investments required [301]. Synthetic data augmentation, generating privacy-preserving synthetic pediatric tumor datasets from small real-world training sets, is a methodologically promising approach requiring validation to ensure synthetic data faithfully represents the biological characteristics of rare pediatric tumors [302]. AI-guided clinical trial matching and eligibility screening for pediatric oncology trials, leveraging EHR and genomic data, represents a near-term implementable application that does not require large imaging datasets and directly addresses the challenge of under enrollment that limits evidence generation in pediatric cancers [303].

#### 3.24.4. Data Source Demographics and Generalizability Risks

A recurring limitation across the oncologic AI literature reviewed in this manuscript is that translational readiness depends not only on model architecture or reported performance, but also on the demographic and geographic composition of the datasets used for development and validation. Across several cancer types, the reviewed evidence highlights important risks to external validity, including breast cancer screening models developed predominantly in White populations, melanoma tools with reduced reliability in darker skin tones, hepatocellular carcinoma models trained largely in viral-etiology cohorts that may not generalize to NASH- or alcohol-related disease, and cervical cancer algorithms whose intended deployment environments differ substantially between high-income settings and low- and middle-income countries. These examples illustrate that strong performance in curated development cohorts does not ensure equivalent performance across ancestry groups, disease phenotypes, imaging platforms, or healthcare systems. Accordingly, demographic representation, geographic diversity of data origin, and subgroup-specific external validation should be regarded as core components of translational readiness rather than secondary implementation considerations.

## 4. Conclusions

**Figure 6:**
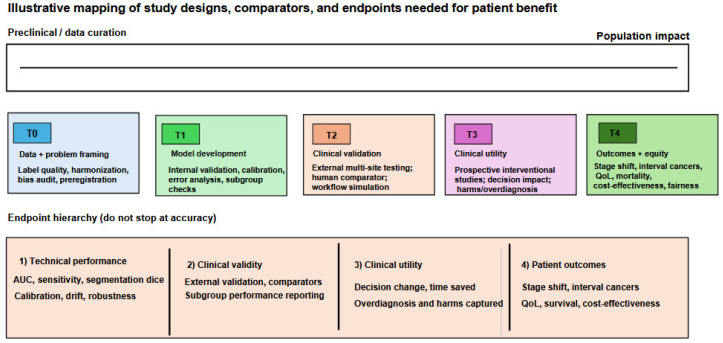
Translational evidence continuum for oncology AI (T0–T4). Mapping of AI development across the translational research spectrum, from data generation and model development to prospective validation, clinical implementation, and population-level impact.

**Figure 7:**
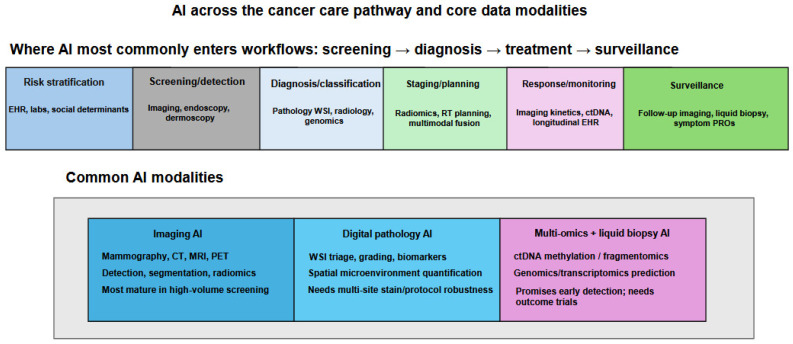
AI across the cancer care pathway and major data modalities. Overview of how AI can support multiple stages of cancer care—including risk stratification, detection, diagnosis, treatment planning, and surveillance—using imaging, pathology, genomic, and clinical data.

**Figure 8:**
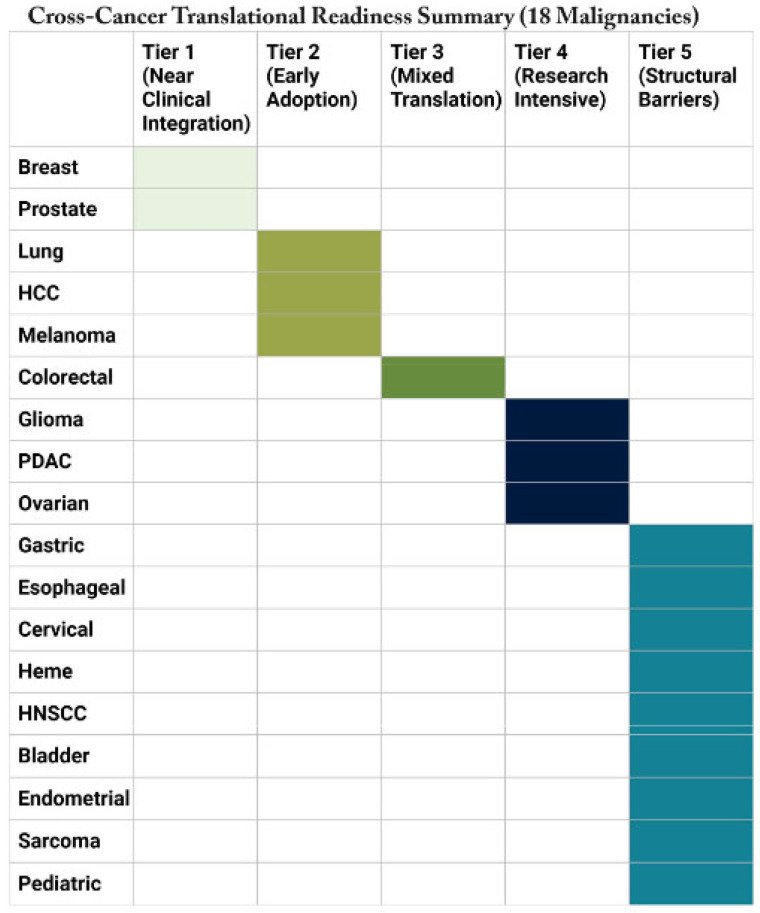
Cross = cancer translational readiness summary. Overview of tier assignment based on the review’s evidence framework.

This review of artificial intelligence across 18 major malignancies establishes that oncological AI, despite its remarkable technical achievements, has not yet fulfilled its clinical promise. Across all five translational tiers (Figure 6), from regulatory-cleared tools in breast and prostate cancer to early feasibility studies in sarcoma and pediatric oncology, a single finding is consistent: diagnostic accuracy is not a surrogate for patient benefit. AI tools with high sensitivity and specificity have repeatedly failed to demonstrate equivalent reductions in cancer-specific mortality, overdiagnosis, or procedural harm when subjected to real-world outcome scrutiny. Simultaneously, documented performance disparities across races, ethnicity, disease etiology, and geographic setting reveal that current AI systems risk amplifying the very health inequities they are positioned to resolve. Bridging this translational gap requires three coordinated shifts: regulatory frameworks that mandate post-market outcome surveillance as a condition of clinical clearance; prospective trial designs that measure patient-centered endpoints rather than diagnostic concordance alone; and sustained infrastructure investment in federated data governance, demographically inclusive training datasets, and LMIC-accessible regulatory pathways (Figure 7). AI holds genuine potential to reduce cancer mortality at a global scale—but only if it is held to the evidentiary and equity standards that the stakes of oncological care demand. A five-tier translational readiness framework, grounded in the NIH T0–T4 translational spectrum and CONSORT-AI/SPIRIT-AI guidelines, was applied a priori to enable cross-cancer comparison (Figure 8) (Table S1). A rigorous distinction was maintained between diagnostic accuracy and clinical utility, defined as demonstrated impact on clinical decision-making or patient-centered outcomes.

## Figures and Tables

**Table 1:** Cross-cutting translational failure modes and mitigation strategies across cancers (manuscript synthesis).

Cross-Cutting Failure Mode	Why It Matters (as Framed in Manuscript)	Illustrative Cancer Contexts Used in Manuscript	Mitigation Direction Described or Directly Implied
Accuracy without clinical utility	Improved diagnostic metrics do not automatically translate to better outcomes; outcome impact is the unresolved question in several domains.	Breast cancer (accuracy vs mortality/interval cancer evidence gap); colorectal cancer (detection gains vs clinically meaningful endpoints); gliomas (unclear when predictions change decisions).	Pre-specify decision points the tool is intended to influence and measure downstream decision and outcome endpoints rather than accuracy alone.
Overdiagnosis amplification	Higher sensitivity can increase procedures and harms if detected lesions are clinically indolent; population benefit may worsen despite better detection.	Prostate cancer (overdiagnosis concerns); lung cancer (nodule detection vs clinical significance); colorectal cancer (adenoma detection without advanced neoplasia improvement).	Shift evaluation to advanced disease endpoints (stage shift, interval cancers) and explicitly capture harms and downstream utilization.
Generalizability failure (protocol/population shift)	Controlled validation differs from real-world deployment; performance degradation occurs with heterogeneous protocols and populations.	Breast cancer (dense breasts and non-European populations); lung cancer (community deployment); HCC (viral vs NASH/alcohol etiologies).	Require external multi-institutional validation; evaluate domain shift; develop multi-center training approaches (including federated methods) and subgroup analyses.
Dataset demographic/phenotype bias	Training imbalances can produce disparate errors across skin tone, ancestry, and disease phenotype; equity risks are explicitly emphasized.	Melanoma (skin tone performance concerns); breast cancer (trial demographic imbalance); HCC (etiology-specific bias).	Representative data collection; pre-specified subgroup reporting; fairness auditing and monitoring as part of readiness claims.
Concept drift (changing disease definitions)	Evolving classifications can make historically trained models systematically incorrect.	Gliomas (WHO 2021 reclassification impact on labels and model validity).	Versioning and drift monitoring; retraining with contemporary labels; explicit scope statements for model applicability.
Workflow incompatibility/partial automation risk	Many cancers require multiparametric workflows; improving one component may not map to decision-making and can mislead if used out of context.	Hematologic malignancies (morphology vs flow/molecular workflow); bladder cancer (surveillance protocol integration).	Embed tools into real clinical systems; evaluate trust, escalation, and error recovery; integrate modalities when the clinical workflow is inherently multimodal.
Governance and post-market evidence gaps	Regulatory clearance or technical success is not equivalent to patient benefit; ongoing monitoring is necessary.	Breast cancer (historical CAD lesson and outcome scrutiny); melanoma (post-market subgroup evaluation requirements discussed).	Post-market surveillance; outcome tracking; equity-stratified reporting; clearly documented intended use and failure modes.

**Table 2:** Modality-to-task mapping and translational readiness patterns across the review (manuscript synthesis).

Modality/Paradigm	Representative Data Types Described	Typical Clinical Tasks Described	Where Translation Is Positioned as Most Mature Within the Review	Common Barriers Emphasized in the Review
Imaging AI (CNNs; radiology/endoscopy vision models)	Mammography; CT; MRI; PET; ultrasound; endoscopy/dermoscopy images and video.	Screening/detection; lesion characterization; segmentation; radiomics-driven prediction; response monitoring concepts.	Most mature where digital infrastructure and standardized workflows exist (e.g., mammography screening; prostate MRI contexts); early adoption in lung CT screening workflows.	Protocol heterogeneity; false positives and overdiagnosis risks; performance degradation in real-world settings; outcome-level evidence gaps.
Digital pathology AI (WSI)	Whole slide images (H&E); pathology workflows; biomarker scoring and metastasis detection contexts described.	Diagnosis/classification; grading; biomarker quantification; metastasis/lymph node detection; molecular inference from morphology described as emerging in multiple cancers.	More mature in cancers with established digitization and benchmarking ecosystems (breast/prostate examples in manuscript).	Stain/protocol variability; difficult borderline cases; unclear incremental utility when molecular testing is already standard in high-resource settings; generalizability across institutions.
Radiomics/radiogenomics	Quantitative features from CT/MRI/PET; segmentation pipelines; feature extraction/selection and ML prediction.	Response prediction; prognostic modeling; molecular association inference (radiogenomics) described across cancers.	Widespread technical maturity across multiple cancers, but often without clear clinical utility evidence (positioned as mixed/early depending on cancer).	Reproducibility and external validation challenges; overfitting; imaging protocol variation; uncertain decision impact without prospective evaluation.
Longitudinal EHR ML (sequence models)	EHR temporal sequences; longitudinal labs and clinical encounter trajectories.	Risk prediction and early identification of individuals at elevated future cancer risk; screening/triage concepts.	Highlighted as promising for PDAC risk prediction and earlier detection concepts.	Actionability gap (what surveillance pathway follows a high-risk score); need prospective embedding into workflows; risk of anxiety/utilization without outcome benefit.
Multi-omics + liquid biopsy ML	ctDNA methylation; fragmentomics; genomic/transcriptomic profiles; multi-analyte biomarkers.	Early detection concepts; response monitoring; recurrence detection; biomarker-guided stratification.	Described as an emerging frontier with potential to address limitations of imaging in some settings (lung and HCC examples emphasized).	Requires prospective validation with patient-centered endpoints; integration into clinical workflows; population- and etiology-specific generalizability.
Federated learning/distributed training	Multi-institution training without centralizing patient-level data.	Enables model development across sites where data sharing is constrained; positioned as a pathway to improve generalizability and equity.	Highlighted as enabling for multi-center training (breast examples) and for multi-national model development in data-concentrated cancers (gastric).	Governance and infrastructure requirements; harmonization constraints; not a substitute for prospective outcome trials.
Foundation models and generative methods (as described)	Large pre-trained backbones (pathology/radiology) and synthetic augmentation concepts.	Multi-task adaptation; potential to reduce data scarcity; synthetic data augmentation described for rare contexts.	Positioned as emerging infrastructure that may help generalizability and scarcity constraints.	Still requires rigorous validation, fairness auditing, and outcome-level evidence; governance and drift concerns remain.
Transformers/LLMs (clinical text)	Clinical notes and oncology narratives; pathology report summarization and trial matching contexts described.	Summarization; eligibility screening; structured extraction from unstructured text in oncology workflows.	Positioned as emerging and supportive rather than definitive decision tools.	Needs workflow evaluation and reliability assessment; risks if outputs are interpreted outside intended scope; requires validation in real clinical operations.

**Table 3:** Translational evidence continuum and endpoint hierarchy used in the review (T0–T4).

Evidence Layer (T0–T4)	What It Represents in This Review	What Studies Typically Report at This Layer	What Is Explicitly Treated as Insufficient	What Is Required to Advance
T0—Data + problem framing	Pre-analytic and design groundwork before model building.	Task definition; data harmonization; label definitions; bias considerations; preregistration language in the methods/framework.	Claims of readiness without transparent dataset/label/bias framing.	Move to model development with internal validation and error analysis.
T1—Model development	Algorithm development and internal testing (technical feasibility).	Internal validation; calibration and error analysis; subgroup checks as described in the framework text; retrospective feasibility evidence predominates across cancers.	High AUC/sensitivity alone, without calibration/robustness and subgroup performance characterization.	Independent external validation using appropriate comparators and realistic workflow simulation.
T2—Clinical validation	External evaluation across sites with clinical comparators.	External multi-site testing; comparator to clinician performance; workflow simulation framing; multi-institutional evaluations described as features of higher readiness.	External accuracy without demonstrated decision impact or harms assessment.	Prospective studies designed to measure clinical utility (decision impact, time, harms/overdiagnosis).
T3—Clinical utility	Evidence that AI changes clinical decisions in practice or trials.	Prospective interventional study designs; endpoints that include decision impact and capture harms/overdiagnosis (framework language).	Inferring utility from performance metrics alone; measuring only concordance rather than downstream consequences.	Outcome-level evaluation (stage shift, interval cancers, QoL, survival) with equity-stratified reporting and governance.
T4—Outcomes + equity	Patient-centered benefit and fairness demonstrated under real-world conditions.	Stage shift; interval cancers; QoL; mortality; cost-effectiveness; fairness (framework text).	Treating diagnostic accuracy as a surrogate for patient benefit; ignoring disparities or real-world performance degradation.	Post-market surveillance and continuous monitoring, including subgroup performance and drift, as a condition for sustained deployment.

Endpoint hierarchy emphasized in the review: technical performance → clinical validity → clinical utility → patient outcomes (including harms/overdiagnosis and equity).

## Data Availability

No new data were created or analyzed in this study. Data sharing is not applicable to this article.

## Supplementary Materials

The following supporting information can be downloaded at: https://www.mdpi.com/article/10.3390/cancers18101543/s1, Table S1: Cross-cancer translational readiness summary across 18 malignancies.
