# Supplementary material for: Artificial Intelligence in Oncology: A Comprehensive Cross-Cancer Translational Readiness Analysis Across 18 Malignancies
Source: Cancers (Basel). 2026 May 10;18(10):1543. doi: 10.3390/cancers18101543 (PMC13204750; doi:10.3390/cancers18101543)
Supplement: Supplementary file 1 [file cancers-18-01543-s001.zip › cancers-4235088-supplementary.pdf]

Artificial Intelligence in Oncology: A Comprehensive Cross-Cancer Translational Readiness Analysis Across 18 Malignancies

Supplementary Table S1. Cross-cancer translational readiness summary across 18 malignancies.

Cancer type	Most mature/prominent AI applications described	Key translational limitations / barriers emphasized	Future development priorities highlighted
Breast cancer	AI-assisted mammography screening with regulatory approval; pathology AI for HER2/Ki-67/hormone receptor expression and lymph node metastasis detection; morphological-based molecular profiling from H&E; federated learning for neoadjuvant response prediction.	Performance degradation in dense breast tissue and non-European populations; pivotal trials predominantly White with subgroup disparities; unresolved outcome impact (interval cancers, stage migration, mortality) and historical caution from prior mammography CAD.	Fairness auditing across breast density and ancestry; integrate mammographic AI risk with polygenic risk scores and lifestyle risk factors; expand multi-national federated learning; consider multimodal integration with liquid biopsy signals.
Prostate cancer	MRI AI for detection of clinically significant prostate cancer within PI-RADS context; pathology AI for Gleason grading and biopsy triage; automated radiotherapy planning feasibility; multimodal models for outcome prediction beyond standard risk stratification.	Overdiagnosis concern (increased detection may include indolent disease); generalizability across imaging systems and practice settings; performance variance in borderline Gleason distinctions that influence treatment decisions.	Prospective trials evaluating whether AI-guided biopsy decisions reduce overdiagnosis while maintaining detection of clinically significant disease; integrate PSMA-PET with MRI-based AI for recurrence risk stratification; AI-guided focal therapy planning.
Lung cancer	Low-dose CT screening: nodule detection tools integrated into workflow; AI-enhanced malignancy risk estimation beyond Lung-RADS; histopathology AI for NSCLC subtype classification and mutation inference; liquid biopsy ML using fragmentomics and methylation profiling for detection/monitoring concepts.	Central challenge is risk stratification rather than detection; overdiagnosis and false positives in community deployment; unclear clinical role of histology-based mutation prediction when molecular testing is available; performance variation by histologic subtype.	Radiomics-pathology fusion for immunotherapy response prediction; adaptive radiotherapy planning using real-time response assessment; subgroup-stratified validation across exposures and ethnicity-associated mutation profiles.
Hepatocellular carcinoma	Imaging AI for lesion detection/characterization on ultrasound/CT/MRI with feasibility for automated LI-RADS classification; cfDNA methylation-based early detection concept; AI fibrosis staging from biopsy WSI; models for microvascular invasion prediction entering prospective validation.	Etiology-specific generalizability gap: models trained on viral etiologies may not generalize to NASH- and alcohol-related HCC; need validation against clinical consequences of LI-RADS categories; rare variants described as failure modes.	Etiology-stratified training/validation with deliberate representation of NASH- and alcohol-related disease; AI-optimized surveillance interval personalization beyond fixed 6-month ultrasound by integrating AFP trajectory, Child-Pugh score, and imaging features.
Melanoma	AI-enabled device authorized for skin cancer detection in primary care; dermoscopy AI with dermatologist-comparable performance in controlled	Specificity remains low in primary care deployment (high false-positive referral burden); demographic bias in training data and performance degradation in	Multi-ethnic training cohorts with representation of Fitzpatrick IV-VI; dermoscopy-pathology fusion to improve specificity; AI-augmented total body

	studies; pathology AI for Breslow depth quantification; histology-based models for sentinel lymph node status and TIL-based immunotherapy associations described.	darker skin tones; need representative data collection and equity-focused validation.	photography for longitudinal surveillance; validate risk stratification models for safely deferring sentinel node biopsy.
Colorectal cancer	CADe during colonoscopy with FDA-cleared systems increasing adenoma detection; CADx for optical polyp diagnosis; pathology AI for MSI prediction and prognostic features such as tumor-stroma ratio and H&E-based prognostic tools.	Clinical utility debate unresolved: CADe increases adenoma detection but not advanced adenomas in meta-analysis; increased removal of nonneoplastic polyps; CADx shows no clear benefit for resect-and-discard; dataset structure limits real-world utility.	Outcome-driven prospective trials (interval cancer rates and CRC incidence over long follow-up); validate MSI prediction against immunotherapy response in real-world cohorts; develop recurrence risk prediction after curative resection integrating pathology, molecular, and imaging features.
Brain tumors (gliomas)	MRI-based radiomics/DL for IDH and 1p/19q inference and grading; intraoperative rapid molecular classification tools within surgical time constraints; feasibility for pseudoprogression versus true progression differentiation.	Prospective outcome validation lacking for decision impact; surgical extent constrained by functional anatomy; concept drift risk due to WHO 2021 reclassification; rare subtypes described as failure modes.	Prospective trials specifying scenarios where AI-predicted molecular status guides decisions; AI-guided surgical margin delineation integrating intraoperative imaging; improved pseudoprogression versus progression models incorporating longitudinal context.
Pancreatic ductal adenocarcinoma	EHR-based risk prediction using longitudinal trajectories; imaging segmentation for pancreas/lesions; radiomics for differentiating PDAC from autoimmune pancreatitis and standardizing resectability assessment concepts.	Absence of validated early detection paradigm; actionable surveillance pathways remain undefined; data scarcity and external validation failures are recurrent barriers.	Prospective studies embedding AI risk stratification into workflows linked to defined surveillance pathways; multi-analyte liquid biopsy discovery/validation; intraoperative imaging integration for surgical planning.
Ovarian cancer	Ultrasound AI for adnexal mass triage feasibility; pathology subtype classification; HRD prediction from H&E described as emerging; multi-omics models for platinum sensitivity and survival described.	Absence of effective early detection paradigm; screening experience described as not demonstrating mortality benefit; histologic heterogeneity and limited prospectively collected multimodal datasets.	Composite early detection in high-risk populations integrating imaging and biomarkers; prospective validation of histology-based HRD prediction against PARP inhibitor response; spatial multi-omics integration with outcomes.
Gastric cancer	Endoscopic real-time detection in high-prevalence East Asian populations; pathology feasibility for Lauren classification and HER2 scoring; exploratory perioperative response prediction described.	External validation outside East Asia is limited; regional biological and epidemiologic differences undermine generalizability.	Multi-regional validation cohorts; federated learning for multi-national training; prospective validation for response prediction before clinical deployment.
Esophageal cancer	Endoscopic AI for dysplastic Barrett's detection and biopsy targeting; squamous dysplasia detection investigation; EUS image analysis for staging feasibility;	Need large prospective studies for surveillance strategies; outcome-level mortality questions require long-term follow-up.	Prospective outcome trials assessing AI-targeted biopsy strategies; adaptive radiotherapy planning using mid-treatment response assessment;

	radiomics for response prediction described.		integration with non-invasive monitoring concepts after definitive therapy.
Cervical cancer	AI-augmented VIA using smartphone-based algorithms; automated cervical cytology; colposcopy image guidance; p16/Ki-67 dual stain interpretation; HPV genotyping integration described.	Generalizability challenges across populations (HPV genotype distribution, prevalence effects, image acquisition quality, HIV-associated ecology); lower performance for glandular lesions; regulatory pathway gaps in LMICs.	LMIC-specific training with validation against clinical outcomes; LMIC-inclusive regulatory frameworks and WHO prequalification pathways; HPV genotyping integration enabling AI-predicted extended genotype risk scores from liquid-based cytology without separate molecular testing.
Hematologic malignancies	Morphologic analysis of blood smears and bone marrow; blast counting; lymphocyte subtype classification; flow cytometry ML for MRD detection described.	Workflow incompatibility with multiparametric diagnostic framework; subtype heterogeneity; dynamic disease biology under therapy affecting generalization.	Integrated multimodal AI combining flow cytometry, morphology, and molecular genetics; AI-guided clonal architecture analysis from single-cell sequencing linked to response and survival endpoints.
Head and neck squamous cell carcinoma	HPV status prediction from H&E described; radiomics for locoregional control prediction; AI-guided adaptive radiotherapy re-segmentation and replanning described.	Small heterogeneous datasets across subsites and HPV strata; protocol heterogeneity limits generalizability.	AI-guided treatment de-escalation decision support for HPV-positive disease using large multi-institution datasets; AI-augmented ctDNA surveillance concepts for post-treatment monitoring.
Bladder cancer	Cystoscopy augmentation including flat lesion/CIS detection feasibility; automated detection of papillary lesions; pathology feasibility for staging and muscularis propria quantification; urine cytology automation investigation.	Workflow integration constraints within surveillance protocols; value proposition depends on clinically significant recurrences detected and false-positive burden.	Urine-based liquid biopsy AI for recurrence monitoring and potential cystoscopy interval extension (if validated); prospective validation against cystoscopy-confirmed recurrence; prospective multi-center validation for enhanced imaging integration during cystoscopy.
Endometrial cancer	AI for predicting molecular subgroup membership from histology; MRI radiomics for lymph node metastasis risk prediction described.	Prospective data scarcity; reliance on retrospective cohorts; need validation against clinical outcome questions, not concordance alone.	Prospective validation linked to adjuvant therapy decisions and survival outcomes; AI integration with minimally invasive biopsy specimens; randomized validation for nodal staging decision support.
Sarcoma	Radiomics feasibility for treatment response assessment; pathology feasibility for diagnostically challenging distinctions; fusion gene status prediction from H&E described as triage concept.	Extreme rarity and heterogeneity; diagnostic concordance challenges; generalizability barriers from limited training cohorts.	International consortium-based data sharing and federated learning; few-shot learning approaches; AI-guided digital pathology consultation tools linking community pathologists to reference centers.
Pediatric solid tumors	Neuroblastoma radiomics risk stratification feasibility; Wilms tumor histologic classification feasibility; Ewing sarcoma	Ethical and regulatory constraints; re-identification risk in small populations;	International consortia-led data harmonization and federated learning; harmonized imaging and annotation;

	triage concepts; pediatric genomic AI described within broader profiling programs.	adult-trained models may fail due to biological distinctions.	synthetic data augmentation with validation; AI-guided clinical trial matching and eligibility screening.
